# Of Clams and Clades: Genetic Diversity and Connectivity of Small Giant Clams (*Tridacna maxima*) in the Southern Pacific Ocean

**DOI:** 10.1002/ece3.70474

**Published:** 2024-10-25

**Authors:** Ryan J. Nevatte, Michael R. Gillings, Kirby Morejohn, Lara Ainley, Libby Liggins, Morgan S. Pratchett, Andrew S. Hoey, Peter C. Doll, Brendon Pasisi, Jane E. Williamson

**Affiliations:** ^1^ School of Natural Sciences Macquarie University Sydney New South Wales Australia; ^2^ ARC Centre of Excellence in Synthetic Biology Macquarie University Sydney New South Wales Australia; ^3^ Ministry of Marine Resources Rarotonga Cook Islands; ^4^ School of Biological Sciences University of Auckland Auckland Aotearoa New Zealand; ^5^ Auckland Museum Tāmaki Paenga Hira Auckland Aotearoa New Zealand; ^6^ College of Science and Engineering James Cook University Townsville Queensland Australia; ^7^ Niue Ocean Wide Niue

**Keywords:** Australia, Cook Islands, Coral Sea, cytochrome oxidase 1, mitochondrial DNA, phylogeography, Tridacninae

## Abstract

Giant clams (*Tridacna* and *Hippopus*) are large marine bivalves occupying tropical and subtropical reefs in the Indo‐Pacific. Giant clam populations have declined in many areas of the Indo‐Pacific and continue to be threatened by harvesting and environmental change. The small giant clam (*Tridacna maxima*) occurs throughout the Indo‐Pacific and has been subject to several phylogeographic studies across its range. However, given its broad range, there are several areas where the genetic diversity and connectivity of *T. maxima* populations has not been characterised. Here, we analyse the mitochondrial marker cytochrome oxidase 1 (CO1) to examine the genetic diversity and connectivity of *T. maxima* in two regions: Australia's Coral Sea Marine Park and the Cook Islands. Samples were collected from 13 reefs within the Coral Sea Marine Park and ten islands within the Cook Islands archipelago. *Tridacna maxima* across the sampled region of the Coral Sea did not display any population structure, whereas significant population structure was detected for *T. maxima* within the Cook Islands. For the Cook Islands, most pairwise comparisons involving an island in the northern group (Manihiki) were significant, as were comparisons for Palmerston (a more centrally located island) and the southern islands, Rarotonga and Mangaia. Both regions displayed high haplotype diversities (> 0.90), indicating that they are important repositories of genetic diversity. Additional CO1 data from throughout *T. maxima*'s distribution showed that the Coral Sea clams belonged to the clade occurring in the South‐Western Pacific Ocean, whilst those from the Cook Islands belonged to a unique clade found in the Central Pacific Ocean. This clade extended from Fiji in the west to French Polynesia in the east and the atolls of Palmyra and Tarawa (Kiribati) in the north. Our assessment of genetic diversity and population structure in these regions will assist with management decisions for the species.

## Graphical abstract

We sequenced the cytochrome oxidase 1 (CO1) gene in small giant clams (*Tridacna maxima*) in Australia's Coral Sea Marine Park and Cook Islands to assess the genetic diversity and connectivity of these populations. Both regions had very high haplotype diversities but differed in their population structures, with no structure detected within the Coral Sea and significant structure in the Cook Islands archipelago. When these regions were placed into a global context with additional CO1 data from across *T. maxima*'s distribution, we found that clams from the Coral Sea belonged to a mitochondrial clade present in the South‐Western Pacific Ocean, whilst those from the Cook Islands were part of a clade unique to the Central Pacific Ocean.

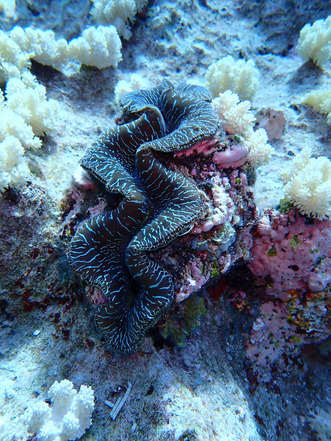

## 1 Introduction

Giant clams (Cardiidae: Tridacninae) are large marine bivalves that occupy reef environments in tropical to subtropical waters of the Indian and Pacific Oceans (Neo et al. 2017). These bivalves make important ecological contributions to reef environments, including the provision of food, shelter and habitat to organisms, filtration of seawater, addition of calcium carbonate to the reef structure and hosting of different phylotypes or species of endosymbiotic dinoflagellates (Neo et al. 2015a; Mies 2019). However, giant clam populations are increasingly under threat from harvesting and environmental change. Harvesting is the main threat, with populations of several species having been overfished in many Indo‐Pacific countries (Lucas 1994). The meat from the clams is used as a food source, whilst the shells can be used and sold as ornaments, sculptures and imitation pearls (Lucas 1994; Larson 2016; Van Wynsberge et al. 2016; Krzemnicki and Cartier 2017). Giant clams are also harvested from the wild to support the global aquarium trade (Vogel and Hoeksema 2024). Beyond harvesting, environmental change, including global warming, ocean acidification, pollution and habitat destruction, also threatens the future of giant clam populations (Mies 2019; Watson and Neo 2021; Sayco, Cabaitan, and Kurihara 2023; Sayco et al. 2024). Due to these threats, all species are listed in Appendix II of the Convention on International Trade in Endangered Species of Wild Fauna and Flora (CITES), and four of the twelve species are listed as Vulnerable on the International Union for the Conservation of Nature (IUCN) Red List of Threatened Species.

The small giant clam (*Tridacna maxima*) is widely distributed throughout the Indian and Pacific Oceans, ranging from the east coast of Africa to the Pitcairn Islands in the Central Pacific (Neo et al. 2017). Previous molecular work on *T. maxima* using mitochondrial DNA has shown that the species comprises several distinct clades (lineages) throughout its range. These clades roughly correspond to particular geographic areas and include three clades in the Western Indian Ocean and Red Sea (Nuryanto and Kochzius 2009; Hui et al. 2016; Fauvelot et al. 2020), two clades in the Central Indo‐Pacific (a North‐Eastern Indian Ocean clade around Java and Sumatra and an Indo‐Malay Archipelago clade encompassing central Indonesia, the Philippines and Taiwan) (Nuryanto and Kochzius 2009; DeBoer et al. 2014; Keyse et al. 2018) and a South‐Western Pacific clade (eastern Australia, New Guinea and Solomon Islands) (Huelsken et al. 2013; Keyse et al. 2018). Several studies also indicate the presence of an additional clade, or possibly two clades, in the Central Pacific (Gardner et al. 2012; Dubousquet et al. 2014; Hui et al. 2016; Keyse et al. 2018; Riquet et al. 2023). The exact geographic extent of *T. maxima* clades in the Central Pacific, however, is currently unknown, as many populations in this region are yet to be investigated. Knowledge of the number of clades present in the region, and their geographic extent, is important for the conservation of *T. maxima*. For example, this information can be used to identify clams that are genetically similar to the resident population for possible restoration programmes, such as captive breeding and reintroduction of adult clams (Frias‐Torres 2017). Such data could also be used to determine the geographic origin of illegally traded giant clam products (Gardner et al. 2012).

Knowledge of genetic diversity and population connectivity at the local (e.g. country) scale is also important for conservation and management. Currently, all IUCN Red List classifications for giant clams, including *T. maxima*, are considered at a global scale and have not been updated since 1996 (Neo et al. 2017). As such, these classifications may not be accurate for populations in individual countries. For example, despite their global assessment as Lower Risk/Conservation Dependent on the IUCN Red List, the population of *T. maxima* in Singapore has been proposed to be Critically Endangered (Neo and Todd 2013). An understanding of local genetic diversity can reveal the potential for a population to respond to change, with more diverse populations having a greater adaptive capacity (Frankham, Ballou, and Briscoe 2010). It can also indicate whether the diversity is spread across an area or concentrated within regions. This can then be used to identify populations vulnerable to possible extinction in the local area (e.g. Dongsha Atoll; Neo et al. 2018) and to assess the appropriateness of the size and placement of marine protected areas in conserving giant clam genetic diversity (Lee et al. 2022).

**FIGURE 1:**
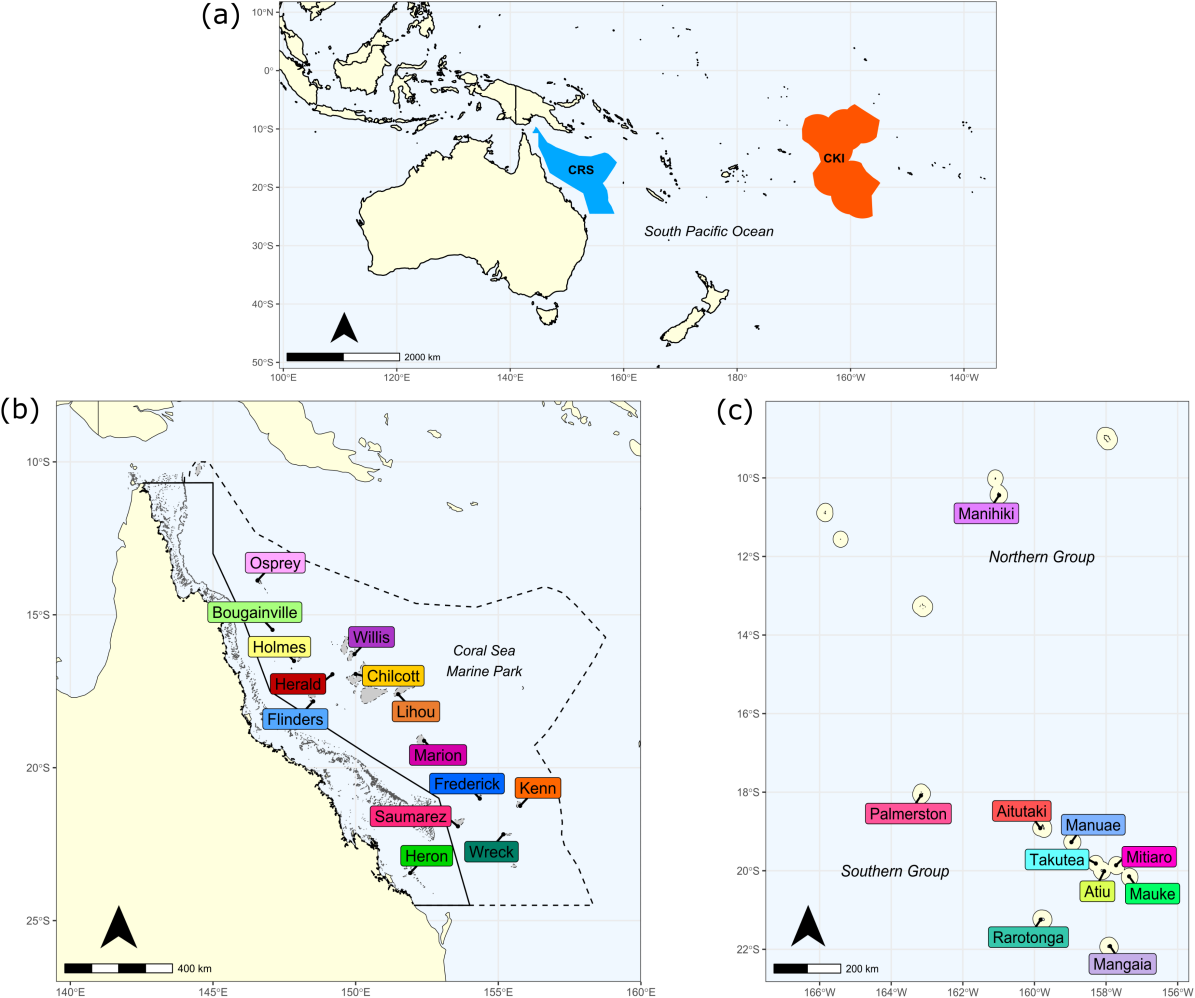
Maps of the regions where *Tridacna maxima* tissue samples were collected in this study. (a) Relative positions of the Coral Sea (CRS) and Cook Islands (CKI) in the South Pacific Ocean. (b) Sites sampled within the Coral Sea, with boundaries of the Coral Sea Marine Park and Great Barrier Reef Marine Park indicated by dotted and solid lines, respectively. (c) Sites sampled within the Cook Islands archipelago. Shapefiles used in (a) and (b) for the Coral Sea were sourced from Australian Marine Parks (http://www.environment.gov.au/fed/catalog/search/resource/details.page?uuid=%7BCD8877F3‐8C39‐4A20‐A53F‐070FBEE5AF3C%7D) and Beaman (2012). Shapefiles used in (a) and (c) for the Cook Islands were sourced from Pacific Data Hub (Cook Islands Exclusive Economic Zone—https://pacificdata.org/data/dataset/exclusive‐economic‐zone‐of‐the‐cook‐islands; Cook Islands Territorial Sea Zone—https://pacificdata.org/data/dataset/cook‐islands‐territorial‐sea‐zone).

Given the wide range of *T. maxima*, many parts of its distribution are understudied. One such region is the Cook Islands, an island country in the central South Pacific Ocean (Figure 1). Six species of giant clam occur in the Cook Islands, namely *T. maxima*, the fluted giant clam (*Tridacna squamosa*), Noah's giant clam (*Tridacna noae*), the smooth giant clam (*Tridacna derasa*), the true giant clam (*Tridacna gigas*) and the bear paw giant clam (*Hippopus hippopus*). Of these six, three are native (*T. maxima*, *T. squamosa* and *T. noae*) (Paulay 1987; Morejohn et al. 2023), whilst the other three species (*T. derasa*, *T. gigas* and *H. hippopus*) were introduced in the late 1980s and early 1990s to support restocking efforts through aquaculture (Sims and Howard 1988; Eldredge 1994). Detailed abundance data for each species are unfortunately lacking, but observational data for the native species indicate that *T. maxima* is the most common and abundant throughout the archipelago, whilst *T. squamosa* and *T. noae* are rarer (Paulay 1987; Sims and Howard 1988; L. Ainley, personal observation). The introduced species are largely restricted to the island of Aitutaki, which also contains a hatchery that produces *T. maxima* and *T. derasa* for restocking of populations on the island (Mies et al. 2017). These restocking operations, however, only operate at a small scale (L. Ainley, personal observation).

Densities of *T. maxima* have declined throughout the Cook Islands, particularly in the southern islands, due to historic overharvesting (Chambers 2007; Morejohn, Ainley, and Kora 2019). Although efforts have been made to protect and restore *T. maxima* (Chambers 2007; Waters, Story, and Costello 2013; Morejohn, Ainley, and Kora 2019), current conservation programmes lack data on genetic diversity and gene flow within the archipelago. Thus far, the only published genetic studies conducted on giant clams in the Cook Islands are an allozyme biogeography study sampling from only one island (Aitutaki) (Benzie and Williams 1997) and a recent study confirming the presence of *T. noae* (Morejohn et al. 2023). Understanding the current genetic diversity and population structure of *T. maxima* in the Cook Islands is critical for developing effective management plans.

Another region that would benefit from knowledge of current genetic diversity and gene flow is Australia's Coral Sea Marine Park (hereafter referred to as Coral Sea) (Figure 1). This is Australia's largest marine park and covers an area of 989,836 km^2^ (Hoey, Pratchett, and Harrison 2020). Seven species of giant clam have been identified within the marine park, including all six species that occur in the Cook Islands and the boring giant clam (*Tridacna crocea*) (Heather et al. 2022). Transect survey data across reefs of the marine park show that *T. maxima* and *T. squamosa* account for ~95% of individuals (Hoey, Pratchett, and Harrison 2020). No restocking programmes are known to operate in the Coral Sea for any of the seven giant clam species, with harvesting pressure on these populations likely to be very low.

Although giant clam populations in Australia, particularly those on the Great Barrier Reef, have received better protection than many other parts of the world, limited population genetics work has been conducted. For *T. maxima*, previous assessments of gene flow within the Coral Sea and Great Barrier Reef have been based primarily on allozymes (Benzie and Williams 1992, 1997). More recent studies using mitochondrial DNA, however, have examined *T. maxima* from a broader biogeographical perspective and, as such, only included a few locations as representatives of the region (Huelsken et al. 2013; Keyse et al. 2018).

Development of effective management plans also relies on accurate identification of the target species. The taxonomy of giant clams has been continually revised and updated since Rosewater (1965), with several species having been newly described or resurrected based on morphological and molecular data (summarised in Tan et al. 2022). Molecular data have been crucial for distinguishing between species due to morphological differences often being subtle, with shell traits and mantle patterns being highly variable, even within species (Su et al. 2014; Johnson et al. 2016; Pappas et al. 2017; Ramesh et al. 2017). This therefore makes in situ identification difficult and is complicated further with some diagnostic traits either not being visible on a live clam (e.g. byssal orifice) or obscured by organisms growing on the shell. Considering habitat ecology, such as whether the species bores into the reef framework or is free‐living on the substrate (Neo et al. 2017, 2019), can assist with identification but these characteristics may not be applicable (e.g. juvenile clams) or easily observed in all instances. Identifying a readily visible morphological feature that aligns with the molecular identity would thus be beneficial for biodiversity surveys involving giant clams.

In this study, we assess the genetic diversity and population structure of *T. maxima* within the Coral Sea (including one site from the Great Barrier Reef) and the Cook Islands using the mitochondrial marker cytochrome oxidase 1 (CO1). This marker has been used in previous population genetics studies on the species throughout its distribution (e.g. Nuryanto and Kochzius 2009; Hui et al. 2016; Lim et al. 2020). Data on genetic diversity and connectivity in these regions will help to inform country‐level IUCN Red List assessments for *T. maxima*. Additionally, genetic diversity metrics between the two regions are compared to assess the effects of legal harvesting on the genetic diversity of *T. maxima* in a harvested (Cook Islands) and protected (Coral Sea) population. The results from these two regions are then placed into a global context using a combination of newly generated and publicly available CO1 data for *T. maxima*. Finally, as CO1 has been effective in differentiating between giant clam species (e.g. Lizano and Santos 2014; Velkeneers et al. 2022), we compare our sequence data with in situ photographs of giant clams to potentially identify a reliable morphological feature that matches the molecular species identity.

## 2 Materials and Methods

### 2.1 Sample Collection

Clam samples from 13 reefs in the Coral Sea (Figure 1b) were collected over the period of 6–23 February 2021 under an Australian Marine Park Activity Permit (Permit Number: PA2020‐00092‐3). Samples were collected opportunistically from clams on the reef crest and slope (~2–12 m water depth) during transect surveys of each reef on SCUBA. Clams were photographed in situ with an Olympus TG‐6 camera before a small piece of mantle tissue was collected. Whilst divers targeted *T. maxima*, given difficulties in differentiating between this species and *T. squamosa* in the field, it is possible that both species may have been sampled during the collection. For sampling, a small implement (for small clams) or wooden block (for larger clams) was first placed between the two valves to prevent the animal from completely closing its shell, and tweezers were then used to pull/stretch the mantle upwards for a small piece of tissue (~0.5 × 0.5 cm) to be cut with scissors. Tissue was placed into 2 mL screw‐cap vials containing 90% ethanol, which were later replaced with 100% ethanol after arriving at Macquarie University. Samples were stored at room temperature. Clam tissue samples from an additional site on the Great Barrier Reef (Heron Island) were collected under permit by M. R. Gillings (Permit Number: GBRMPA 14/3692.1) and preserved as described above.

Tissue samples of clams from ten islands in the Cook Islands (Figure 1c) were collected in 2019 and 2020 following the methods outlined in Morejohn et al. (2023). Briefly, a 1 cm^2^ piece of mantle tissue was collected from clams with haemostats and scissors and placed into 4 mL vials containing 100% ethanol. Samples were collected during reef walks or on snorkel and SCUBA in depths ranging from 0 to 18 m. Sampling specifically targeted wild populations of giant clams on each island, although a few samples (*n* = 8) were collected from clams in the hatchery and nursery areas of Aitutaki. Tissue samples were imported into Australia under a CITES permit (Permit Number: PWS2020‐AU‐001494). On arrival at Macquarie University, the samples had their ethanol replaced with fresh 100% ethanol and were stored at room temperature.

### 2.2 DNA Extraction, PCR and Sequencing

Genomic DNA was extracted from approximately 20 mg of tissue using a modified salting‐out procedure (Sunnucks and Hales 1996) as described in Nevatte et al. (2021). Preliminary trials with these extractions indicated that the samples would not amplify reliably during polymerase chain reaction (PCR), likely due to the co‐purification of PCR inhibitors. Such a problem is not uncommon with mollusc tissue (Adema 2021). Consequently, the DNA extractions were purified further using the Monarch PCR and DNA Cleanup Kit (New England BioLabs) following the manufacturer's instructions. We purified 90 μL of the original extraction with the kit and eluted DNA from the spin columns with 60 μL of DNA elution buffer (pre‐heated to 50°C). These purified extractions were used in all subsequent PCRs.

PCR was used to amplify a 600 base pair (bp) fragment of the mitochondrial CO1 gene with the primer pair SQUAF3 (5′—CATCGTTTAGAGTAATAATTCG—3′) and SQUAR1 (5′—ATGTATAAACAAAACAGGATC – 3′) (DeBoer et al. 2012). Amplification was also attempted with the primer pair CO1‐Tricro‐Frwd and CO1‐Tricro‐Rev (Kochzius and Nuryanto 2008), but this pair did not amplify reliably. PCRs were conducted in 50 μL reaction volumes, with 25 μL of GoTaq Colourless Master Mix (Promega Corporation), 1.5 or 2.5 μL of 50 mM magnesium chloride (for final concentrations of 3 and 4 mM, respectively), 1 μL of 1 mg/mL RNAse A, 0.5 μL of the SQUA primers (50 μM concentration) and 1 or 2 μL of DNA. Nuclease‐free water comprised the remaining reaction volume. Clam samples that failed to amplify in the first instance generally amplified when using the higher magnesium concentration and DNA volume. Cycling conditions consisted of an initial denaturation at 94°C for 3 min, followed by six cycles of 94°C for 30 s, 45°C for 30 s and 72°C for 1 min, and then 36 cycles of 94°C for 30 s, 51°C for 30 s and 72°C for 1 min. A final extension step at 72°C for 5 min was then performed, with PCR products being held at 4°C at the completion of the programme.

Amplification success was determined using gel electrophoresis. Five microlitres of the PCR products were electrophoresed on 1.5% agarose‐TBE gels run at 85 V for approximately 50 mins. Gels were post‐stained with GelRed (Biotium) and photographed under UV‐transillumination. A 100 bp ladder (Invitrogen, Thermo Fisher) was included in all gels to ensure that amplicons were of the correct size.

PCR products were sent to Macrogen (Seoul, South Korea) for purification and Sanger sequencing. The first batch of PCR products were sequenced with both the SQUAF3 and SQUAR1 primers, but sequences produced with SQUAR1 were of poor quality. Thus, all subsequent sequences were produced with the SQUAF3 primer only.

### 2.3 Initial Sequence Analysis

DNA sequences from Macrogen were imported into Geneious Prime 2022.2.2 (www.geneious.com) for initial sequence analysis. Sequences were first trimmed to remove the SQUAR1 primer sequence from the 3′ end and poor‐quality reads from the 5′ end. The remaining sequence was then inspected for nucleotide assignment errors and edited where necessary, resulting in a final sequence of 421 bp for *T. maxima* and 455 bp for all other species. Given the potential for misidentification in the field, initial species identity based on photographs was checked by comparing the trimmed clam sequences with entries in NCBI GenBank using the nucleotide Basic Local Alignment Search Tool (BLAST) (Altschul et al. 1990; Zhang et al. 2000). Sequences were then grouped into separate datasets by species, aligned with the MUSCLE alignment algorithm (Edgar 2004) in Geneious Prime, and collapsed into haplotypes with DnaSP Version 6.12.03 (Rozas et al. 2017). Haplotypes were translated into proteins using the online EMBOSS Transeq tool (https://www.ebi.ac.uk/jdispatcher/st/emboss_transeq) (Madeira et al. 2024) with the invertebrate mitochondrial DNA genetic code to ensure the sequencing of genuine mitochondrial genes. Any individuals with haplotypes that translated into non‐functional proteins (i.e. contained stop codons in the middle of the sequence) with the three forward reading frames were removed from the dataset. Individuals with poor‐quality sequence reads were also removed.

### 2.4 Genetic Diversity and Population Structure

Genetic diversity and population structure in *T. maxima* sampled from the Coral Sea (including the Heron Island clams) and Cook Islands was assessed using Arlequin Version 3.5.2.2 (Excoffier and Lischer 2010). Metrics of genetic diversity, including haplotype (h) diversity and nucleotide (π) diversity, were calculated for each sampled site and each region (either Coral Sea or Cook Islands). Population structure within each region was assessed through an analysis of molecular variance (AMOVA) (Excoffier, Smouse, and Quattro 1992), which tested the null hypothesis of panmixia across sampled locations using two *F*‐statistic metrics, *F*_ST_ (Wright 1965) and *Φ*_ST_ (Excoffier, Smouse, and Quattro 1992). *Φ*_ST_ was calculated by the computation of a distance matrix with the best‐fitting nucleotide substitution model identified by MEGA11 (Tamura, Stecher, and Kumar 2021) for each dataset. The Tamura 3‐parameter model (Tamura 1992) was identified as the best fit for both datasets based on the Bayesian Information Criterion, with the inclusion of a gamma parameter (G) for the Coral Sea (*G* = 0.14) and inclusion of both G and invariant sites (I) for the Cook Islands (0.89 and 0.71, respectively). As Arlequin does not have an option to input I into the analysis, however, this value was not included for the Cook Islands. Significance of the AMOVA was assessed at *α* = 0.05 following 20,000 permutations.

Genetic differentiation between sampled sites was assessed by pairwise comparisons of *F*‐statistics. Pairwise *F*_ST_, *Φ*_ST_ and associated *p*‐values for each site were calculated in Arlequin with 20,000 permutations. To account for multiple comparisons, the raw *p*‐values were adjusted with the p.adjust function in R (R Core Team 2022) using the Benjamini and Hochberg (1995) procedure. Adjusted *p*‐values < 0.05 were considered statistically significant (Wright 1992). All project files for the Arlequin analyses were created with DnaSP.

Visual assessment of potential population structure was achieved through the generation of haplotype networks. Two median‐joining networks (Bandelt, Forster, and Röhl 1999) were generated in PopART Version 1.7 (Leigh and Bryant 2015): the first comprising sites within the Coral Sea, and the second comprising sites within the Cook Islands. A median‐joining network of the two regions combined was also constructed to identify possible gene flow between *T. maxima* in the Coral Sea and Cook Islands. The Epsilon value for generating the networks was set to 0 to reduce complexity.

Given the high haplotype diversity detected (see Results), two additional median‐joining networks for each region were constructed as described above after removing the third codon position. This was done to reduce the number of haplotypes, and therefore the complexity of the haplotype networks, to enable easier viewing of the main geographic patterns. As the EMBOSS Transeq tool showed that the 421 bp alignment was translating in the second reading frame, the first nucleotide in the alignment was deleted so that the sequence was translating in the first reading frame (420 bp). The third codon position was then stripped from the alignment with the Mask Alignment tool in Geneious Prime, resulting in a final alignment length of 280 bp.

### 2.5 Haplotype Rarefaction Curves

To determine whether the present sampling effort was sufficient to characterise the full genetic diversity of the Coral Sea and Cook Islands populations, haplotype accumulation/rarefaction curves were constructed. For each region, the number of individuals possessing each of the identified haplotypes (i.e. abundance) was determined and then used to generate rarefaction‐extrapolation (R‐E) curves with the R package iNEXT Version 3.0.0 (Chao et al. 2014; Hsieh, Ma, and Chao 2016). Haplotype diversity R‐E curves were calculated using 500 bootstrap replicates for the 95% confidence intervals and 300 knots. The endpoint value for extrapolation was left at the default of twice the current sample size. The calculated Chao1 index was used to estimate the asymptote of the R‐E curve.

### 2.6 Population Expansion

Population expansion in *T. maxima* sampled from the Coral Sea and Cook Islands was tested in DnaSP through neutrality indices and mismatch distributions. For each region, Tajima's *D* (Tajima 1989), Fu's *F*_S_ (Fu 1997) and Ramos‐Onsins and Rozas' *R*_2_ (Ramos‐Onsins and Rozas 2002) were calculated, with the significance of these estimates being tested based on 10,000 simulated samples. Indices were considered statistically significant at *α* = 0.05 for both *D* and *R*_2_, and at *α* = 0.02 for *F*_S_ (Fu 1997). Additionally, mismatch distributions of the observed pairwise differences between sequences and the expected values under a model of constant population size and population growth‐decline were generated to visually assess population expansion. Harpending's raggedness index (H_RI_) (Harpending 1994) was also calculated as a statistical measure of the fit of the data to an expanding population model. Significance of H_RI_ was determined at *α* = 0.05 following 10,000 simulated samples.

### 2.7 Global Population Structure

Additional *T. maxima* CO1 sequences from other parts of its distribution were sourced from two publicly available online databases: the Genomic Observatories Metadatabase (GEOME; https://geome‐db.org/), which provides georeferenced sequence data (Deck et al. 2017; Riginos et al. 2020), and NCBI GenBank. Sequences registered in GEOME included those from the studies of Nuryanto and Kochzius (2009), DeBoer et al. (2014), Keyse et al. (2018) and Huelsken et al. (2013), which covered clades in the Central Indo‐Pacific, Western Pacific and Red Sea. Additional sequences from Fauvelot et al. (2020) for the clades in the Western Indian Ocean and Red Sea were sourced from GenBank.

To expand the number of locations within the Central Pacific, we sourced previously published and unpublished sequences. These included published sequences from Tarawa Atoll (Republic of Kiribati) and Palmyra Atoll [Gardner et al. 2012; obtained from the supplementary material of Keyse et al. 2018] and French Polynesia (Dubousquet et al. 2014; Riquet et al. 2023) and unpublished sequences from Niue, Beveridge Reef, Minerva Reef (Tonga) and Fiji (Liggins and Arranz 2018). For the sequences from French Polynesia (GenBank accessions MF167466–167524), the number of individuals possessing each accession was based on the frequencies reported in Riquet et al. (2023). Accessions either not listed or had a frequency of 0 in Riquet et al. (2023) were given a frequency of one as these were listed in Dubousquet et al. (2014), but no further frequency information was provided.

All additional sequences were imported into Geneious Prime and aligned with the Coral Sea and Cook Islands sequences using the Geneious alignment algorithm. The alignment was then trimmed to a common length of 316 bp. Any sequences within this alignment that contained one or more unknown nucleotides (i.e. N) were removed, whilst sequences containing an ambiguous base (i.e. R) were retained. Single sequences from a location that could not be easily grouped with another location based on the GEOME metadata were also removed. Sequences were then collapsed into haplotypes with DnaSP, specifying that sites containing gaps or missing data were ‘Not considered’ in the definition of the haplotype. This was to avoid artificially inflating the number of haplotypes within the dataset.

To visualise relationships between locations in the Coral Sea and Cook Islands with the previously identified clades, a median‐joining haplotype network of this global dataset was generated in PopART (Epsilon value set to 0 to reduce complexity). The net average genetic distance between each haplogroup/clade in the network was also calculated using the uncorrected P‐distance in MEGA11. Variance was estimated with 100 bootstrap replicates and default settings for all other parameters.

Three pairwise differentiation metrics were calculated for each site in the global dataset, including *F*_ST_, *Φ*_ST_ and Jost's D (Jost 2008). The two *F*‐statistics were calculated in Arlequin with 20,000 permutations, with *Φ*_ST_ calculated using the Tamura 3‐parameter model (Tamura 1992) and a Gamma parameter of 0.294 (identified as the best‐fitting nucleotide substitution model based on the Bayesian Information Criterion in MEGA11). Jost's D was calculated with 1000 bootstraps in SPADE (Chao and Shen 2010), with each haplotype considered as an allele. Sites within the Cook Islands and Coral Sea were not combined for pairwise comparisons (i.e. each island/reef was compared in the analysis). Three sites containing one or two individuals, namely Marion Reef, Mauritius and Bootless Bay (Papua New Guinea), were excluded from the analysis.

Pairwise values for the three metrics were used to test for isolation‐by‐distance (IBD) (Wright 1943) in *T. maxima*. Geographic distances between each site were calculated with the Imap Version 2.01 R package (Wallace 2017) and then regressed against the pairwise values for each metric. Mantel tests (Mantel 1967) were used to assess the relationship between the pairwise geographic and genetic distance matrices with the mantel.randtest function (999 permutations) in the R package ade4 Version 1.7–18 (Chessel, Dufour, and Thioulouse 2004). Permutation tests with the R package lmPerm Version 2.1.0 (Wheeler and Torchiano 2016) were used to calculate the correlation coefficient and assess the significance of the correlation (10,000 permutations; lmp function with the ‘Exact’ method). IBD analyses were performed on both the global dataset and a subset comprising only sites in the Central Pacific.

## 3 Results

**FIGURE A1:**
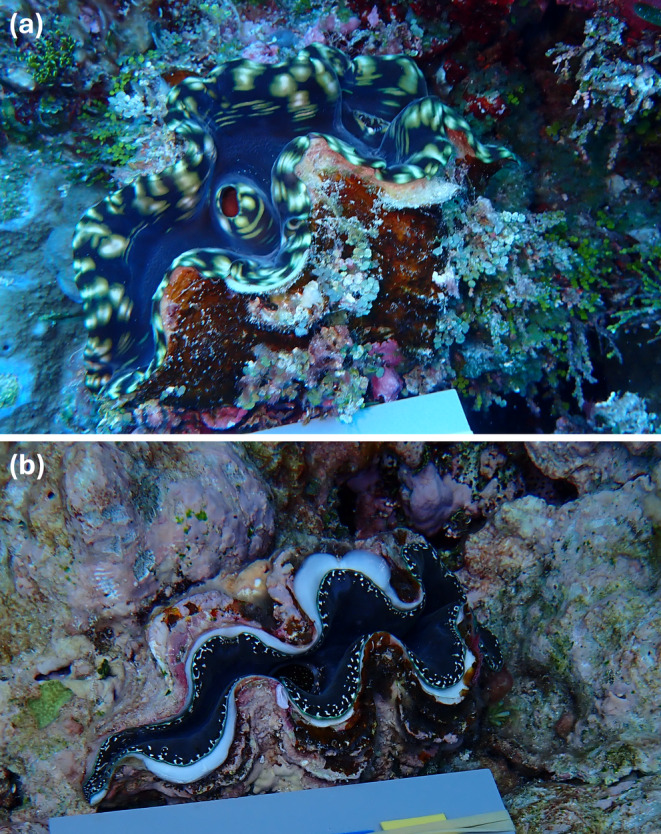
(a) *Tridacna squamosa* (diameter 260 mm) on Flinders Reef (Coral Sea) with matching field identification and genetic identity; (b) Individual clam (diameter 180 mm) from Frederick Reef (Coral Sea) initially identified based on photographs as *Tridacna maxima* but possessed *T. squamosa* CO1. Photo credits: Peter C. Doll.

Sequencing of the CO1 gene for clams from the Coral Sea and Cook Islands confirmed that the majority of clams sampled were *Tridacna maxima* (312 out of 327 and 267 out of 270 individuals, respectively). The limited number of clams sampled from the Coral Sea that were not *T. maxima* included individuals of *Tridacna squamosa* (*n* = 7) (Figure A1a), *Tridacna derasa* (*n* = 7) and *Hippopus hippopus* (*n* = 1). The remaining three individuals from the Cook Islands were *T. squamosa*. Of the eight individuals sampled in the Aitutaki hatchery and nursery, seven were *T. maxima* and one was *T. squamosa*.

From the in situ photographs of the Coral Sea samples, all individuals identified as *T. derasa* produced CO1 sequences matching this species in GenBank, and an individual tentatively identified as being from the *Hippopus* genus produced a CO1 match to *H. hippopus*. Distinguishing *T. maxima* and *T. squamosa* in the field was more difficult. Many individuals for which identification was not certain (i.e. *T. maxima* / *T. squamosa*; *n* = 153), as well as most of those identified as *T. squamosa* (*n* = 45), were subsequently identified as *T. maxima* based on CO1 (153 and 39, respectively). Photographic identification of *T. maxima*, however, was largely successful as most individuals initially assigned as *T. maxima* produced corresponding CO1 sequences. Only one individual was misidentified and possessed a *T. squamosa* CO1 sequence (Figure A1b). All specimens from the Cook Islands produced CO1 sequences that matched their in situ species designation.

### 3.1 Genetic Diversity and Population Structure

Metrics of genetic diversity were similar for the two regions. Overall haplotype diversity (h) for the Coral Sea and Cook Islands was calculated as 0.9357 and 0.9048, respectively, whilst nucleotide diversity (π) for the Cook Islands (0.0082) was slightly lower compared to the Coral Sea (0.0091) (Table 1). Within each region, haplotype diversity ranged from 0.7 to 1 across sampled locations in the Coral Sea and from 0.6980 to 0.9577 in the Cook Islands (Table 1). A wide range for nucleotide diversity was also observed for locations within the two regions (Coral Sea: π = 0.0057–0.0143; Cook Islands: π = 0.0048–0.0127; Table 1).

**TABLE 1:** Genetic diversity indices for *Tridacna maxima* from each of the sampled locations within the Coral Sea and Cook Islands.

Region	Location	*N*	*N*_H_	*N*_UH_	h (±SD)	π (±SD)
*Coral Sea*	Heron Island	5	3	2	0.7000 (±0.2184)	0.0057 (±0.0043)
	Saumarez Reef	15	13	5	0.9714 (±0.0389)	0.0076 (±0.0047)
	Wreck Reef	30	19	12	0.9264 (±0.0340)	0.0099 (±0.0056)
	Kenn Reef	29	18	9	0.9335 (±0.0308)	0.0098 (±0.0056)
	Frederick Reef	22	11	4	0.8831 (±0.0471)	0.0079 (±0.0047)
	Marion Reef	2	2	1	1.0000 (±0.5000)	0.0143 (±0.0154)
	Lihou Reef	34	20	8	0.9376 (±0.0267)	0.0069 (±0.0041)
	Chilcott Reef	10	7	3	0.8667 (±0.1072)	0.0108 (±0.0066)
	Willis Reef	25	18	7	0.9567 (±0.0288)	0.0091 (±0.0053)
	Herald Reef	49	31	18	0.9532 (±0.0189)	0.0090 (±0.0051)
	Flinders Reef	28	18	8	0.9312 (±0.0330)	0.0104 (±0.0059)
	Holmes Reef	7	5	2	0.8571 (±0.1371)	0.0066 (±0.0045)
	Bougainville Reef	27	19	13	0.9402 (±0.0334)	0.0096 (±0.0055)
	Osprey Reef	29	23	10	0.9754 (±0.0182)	0.0104 (±0.0059)
	All locations	312	127	N/A	0.9357 (±0.0087)	0.0091 (±0.0051)
*Cook Islands*	Aitutaki	49	24	8	0.8622 (±0.0470)	0.0063 (±0.0038)
	Atiu	30	17	6	0.8184 (±0.0731)	0.0073 (±0.0043)
	Manihiki	27	12	9	0.6980 (±0.0994)	0.0048 (±0.0031)
	Manuae	29	18	5	0.9015 (±0.0479)	0.0080 (±0.0047)
	Mitiaro	8	6	1	0.8929 (±0.1113)	0.0083 (±0.0054)
	Mauke	10	6	1	0.8444 (±0.1029)	0.0070 (±0.0045)
	Mangaia	6	5	2	0.9333 (±0.1217)	0.0127 (±0.0082)
	Palmerston	28	18	10	0.9577 (±0.0205)	0.0071 (±0.0043)
	Rarotonga	41	24	13	0.9098 (±0.0323)	0.0078 (±0.0045)
	Takutea	39	28	16	0.9528 (±0.0245)	0.0096 (±0.0054)
	All locations	267	101	N/A	0.9048 (±0.0154)	0.0082 (±0.0046)

Abbreviations: *N* = number of individuals; *N*_H_ = number of haplotypes; *N*_UH_ = number of haplotypes unique to the location; h = haplotype diversity; π = nucleotide diversity; N/A = metric not applicable.

**FIGURE 2:**
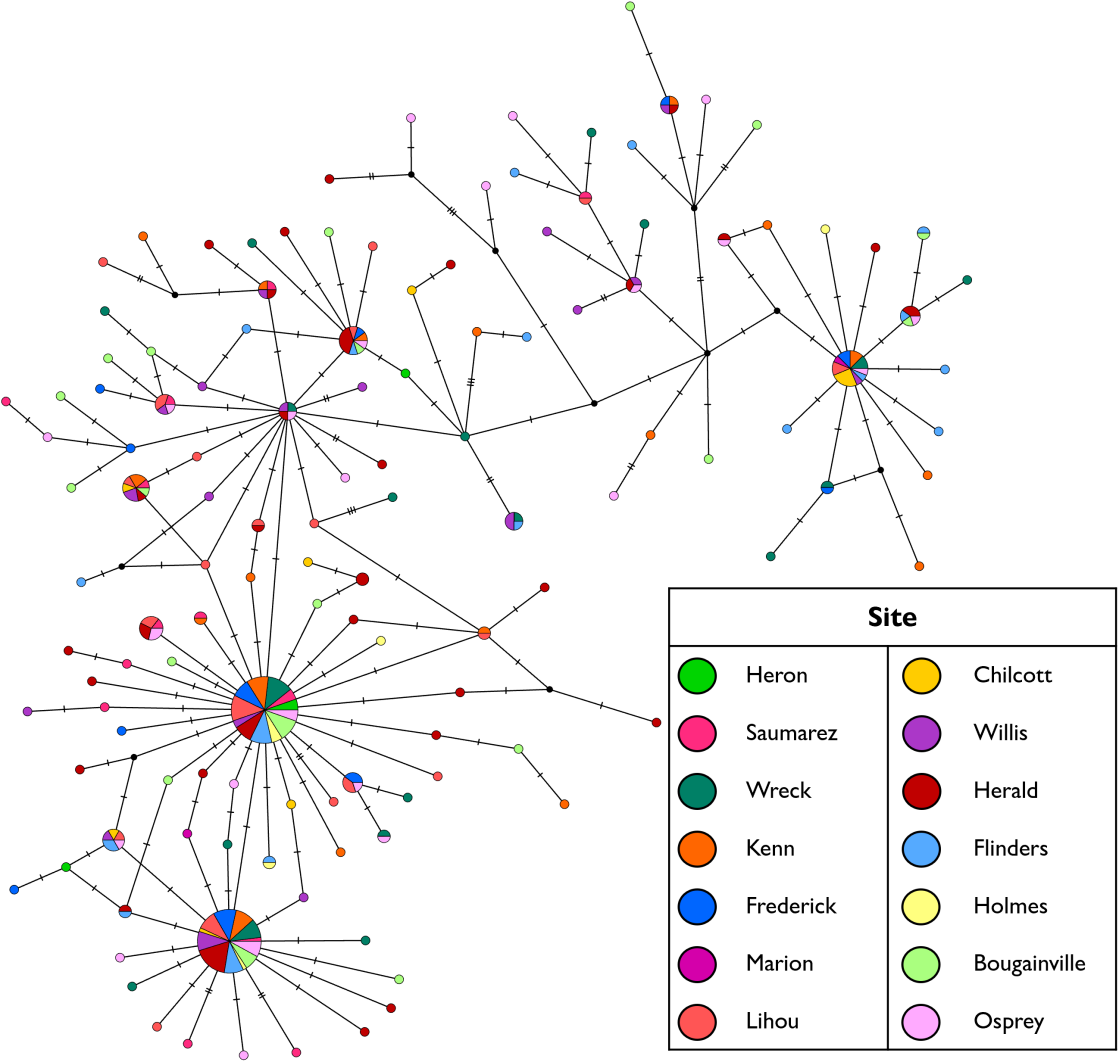
Median‐joining haplotype network for *Tridacna maxima* collected from sites in the Coral Sea. Circle size is proportional to the frequency of each haplotype and colour shading indicates the site where the haplotype was identified. Mutational steps between haplotypes are denoted by hatch marks and small black dots represent hypothetical unsampled haplotypes.

Analysis of molecular variance provided no evidence of significant population structure for *T. maxima* in the Coral Sea. Both F‐statistic metrics (*F*_ST_ and *Φ*_ST_) showed non‐significant *p*‐values, with most of the variation found within sampled locations (Table 2). Thus, the null hypothesis of panmixia could not be rejected for this region. Pairwise *F*_ST_ and *Φ*_ST_ values were also non‐significant between all sites (Table S1). The median‐joining network of the 127 identified haplotypes reflected this lack of structure with no clear separation of haplotypes based on collection site (Figure 2). A few frequently occurring haplotypes were possessed by individuals from most of the 13 reefs sampled. The majority of the identified haplotypes, however, were singletons or haplotypes of low frequency and were separated from their most closely related haplotype by no more than three mutations (Figure 2).

**TABLE 2:** Summary of the analysis of molecular variance (AMOVA) analyses performed on *Tridacna maxima* samples collected in two regions of the South Pacific Ocean.

Region	Metric	Source of variation	df	Sums of squares	Variance components	Percentage of variation	Fixation index
*Coral Sea*	*F*_ST_	Amongst locations	13	6.277	0.00072	0.15	*F*_ST_ = 0.0015; *p* = 0.3393
		Within locations	298	139.223	0.46719	99.85	
	*Φ*_ST_	Amongst locations	13	26.067	0.00318	0.16	*Φ*_ST_ = 0.0016; *p* = 0.3664
		Within locations	298	576.903	1.93592	99.84	
*Cook Islands*	*F*_ST_	Amongst locations	9	7.605	0.01569	3.45	*F*_ST_ = 0.0345; *p* = < 0.0001
		Within locations	257	112.740	0.43868	96.55	
	*Φ*_ST_	Amongst locations	9	55.441	0.17647	9.99	*Φ*_ST_ = 0.0999; *p* = < 0.0001
		Within locations	257	408.602	1.58989	90.01	

**FIGURE 3:**
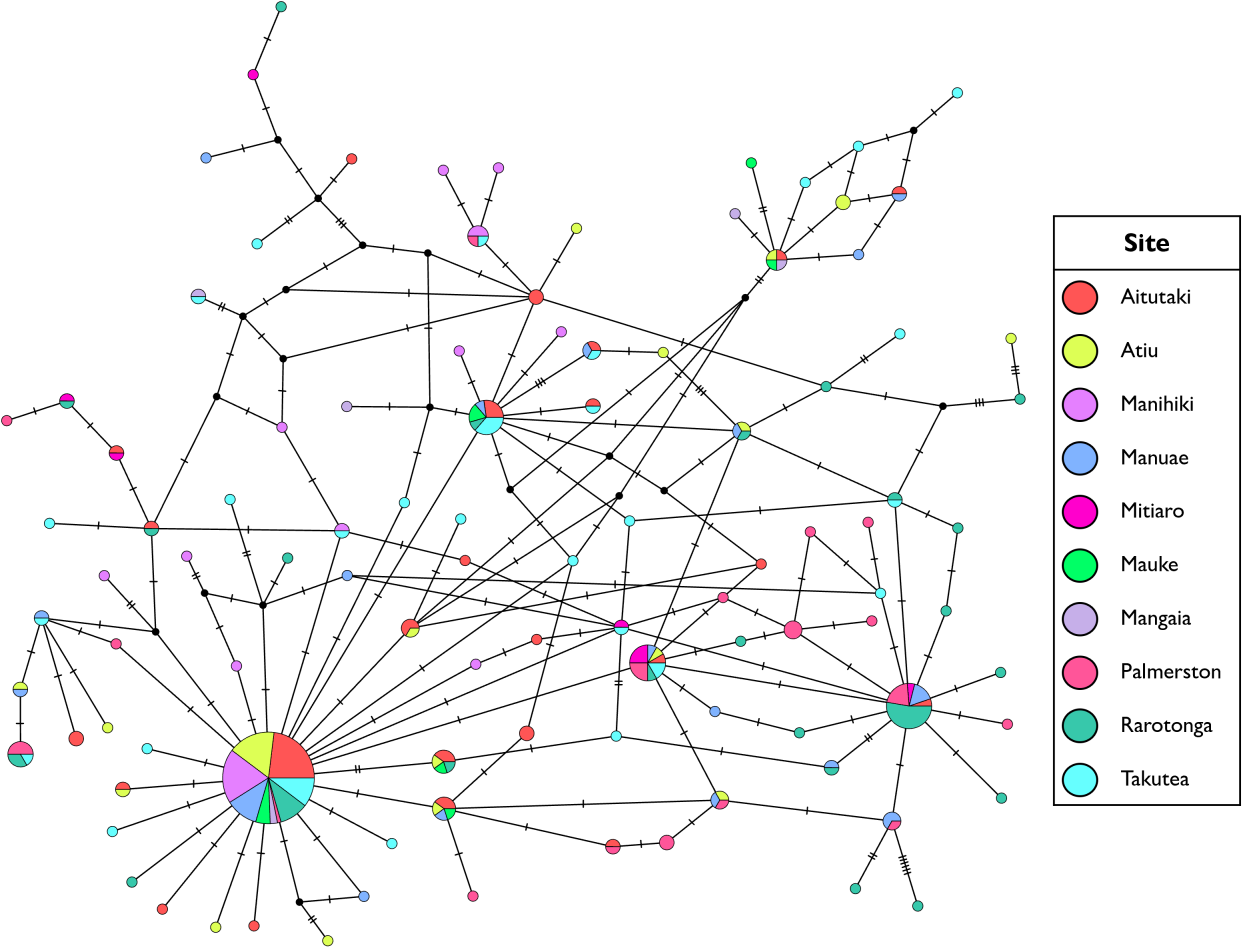
Median‐joining haplotype network for *Tridacna maxima* collected from sites in the Cook Islands. Circle size is proportional to the frequency of each haplotype and colour shading indicates the site where the haplotype was identified. Mutational steps between haplotypes are denoted by hatch marks and small black dots represent hypothetical unsampled haplotypes.

In contrast, analysis of molecular variance for *T. maxima* in the Cook Islands showed evidence for weak but significant population structure. Significant *p*‐values were obtained for both *F*_ST_ and *Φ*_ST_ (Table 2), thus rejecting the null hypothesis of panmixia within the archipelago. Both pairwise *F*_ST_ and *Φ*_ST_ detected significant structuring between sampled locations, with the strongest differentiation being identified with *Φ*_ST_ (Table 3). Most pairwise comparisons involving Manihiki, Palmerston, Rarotonga and Mangaia were significant (Table 3). The median‐joining network of 101 haplotypes largely comprised of singleton or low frequency haplotypes, which were separated from their most closely related haplotype by one to six mutations (Figure 3). The most frequently occurring haplotype (bottom‐left of Figure 3) was identified in 78 individuals across all sites except Mitiaro. For the seven *T. maxima* sampled in the Aitutaki hatchery and nursery, only one possessed a novel haplotype. The remaining six shared haplotypes with other wild sampled individuals.

**TABLE 3:** Pairwise *F*_ST_ values (lower diagonal) and *Φ*_ST_ values (upper diagonal) for *Tridacna maxima* samples collected from the Cook Islands.

	AIT	ATU	MHX	MAN	MIT	MKE	MNG	PAL	RAR	TAK
**AIT**	—	−0.0053	*0.0213*	**0.0362**	**0.2023**	0.0086	**0.1286**	**0.2315**	**0.1584**	0.0054
**ATU**	−0.0070	—	**0.0387**	0.0211	**0.1653**	−0.0110	0.0812	**0.2085**	**0.1407**	0.0002
**MHX**	0.0176	0.0011	—	**0.1103**	**0.3001**	*0.0716*	**0.1877**	**0.3132**	**0.2228**	**0.0313**
**MAN**	−0.0038	−0.0001	*0.0332*	—	0.0479	*0.0698*	**0.1505**	**0.0741**	0.0218	0.0132
**MIT**	**0.1139**	**0.1398**	**0.2240**	*0.0784*	—	**0.2632**	**0.2285**	0.0507	0.0074	**0.1032**
**MKE**	−0.0281	−0.0171	0.0151	−0.0107	**0.1323**	—	−0.0255	**0.2996**	**0.2127**	−0.0031
**MNG**	−0.0216	−0.0220	0.0198	−0.0211	0.0880	−0.0425	—	**0.3540**	**0.2737**	0.0325
**PAL**	**0.0742**	**0.0940**	**0.1526**	**0.0385**	0.0143	**0.0800**	0.0412	—	0.0194	**0.1696**
**RAR**	**0.0370**	**0.0528**	**0.0946**	0.0057	*0.0573*	0.0375	0.0163	0.0179	—	**0.0991**
**TAK**	0.0099	0.0254	**0.0622**	0.0021	*0.0520*	−0.0057	−0.0183	**0.0288**	**0.0242**	—

*Note:* Values in italics were significant at *α* = 0.05 prior to correction for multiple comparisons, whilst values in bold remained significant after the multiple comparison correction.

Abbreviations: AIT = Aitutaki; ATU = Atiu; MAN = Manuae; MHX = Manihiki; MIT = Mitiaro; MKE = Mauke; MNG = Mangaia; PAL = Palmerston; RAR = Rarotonga; TAK = Takutea.

The haplotype network of the two regions combined demonstrated that *T. maxima* in the Coral Sea and Cook Islands are distinct populations (Figure S1). There was no evidence of gene flow as none of the identified haplotypes were shared between the two regions. *Tridacna maxima* in the Coral Sea and Cook Islands formed genetically distinct lineages, which were separated by a minimum of 26 mutations (Figure S1).

Removal of the third codon position resulted in a large reduction in the number of haplotypes for the two regions. The Coral Sea was reduced from 127 to 17 haplotypes and the Cook Islands was reduced from 101 to 23 haplotypes. Both regions produced very similar haplotype networks (Figures S2 and S3), with one central haplotype possessed by most individuals and a few singleton or lower frequency haplotypes. Thus, much of the haplotype diversity exhibited by *T. maxima* was due to nucleotide substitutions at the third codon position.

### 3.2 Haplotype Rarefaction Curves

**FIGURE 4:**
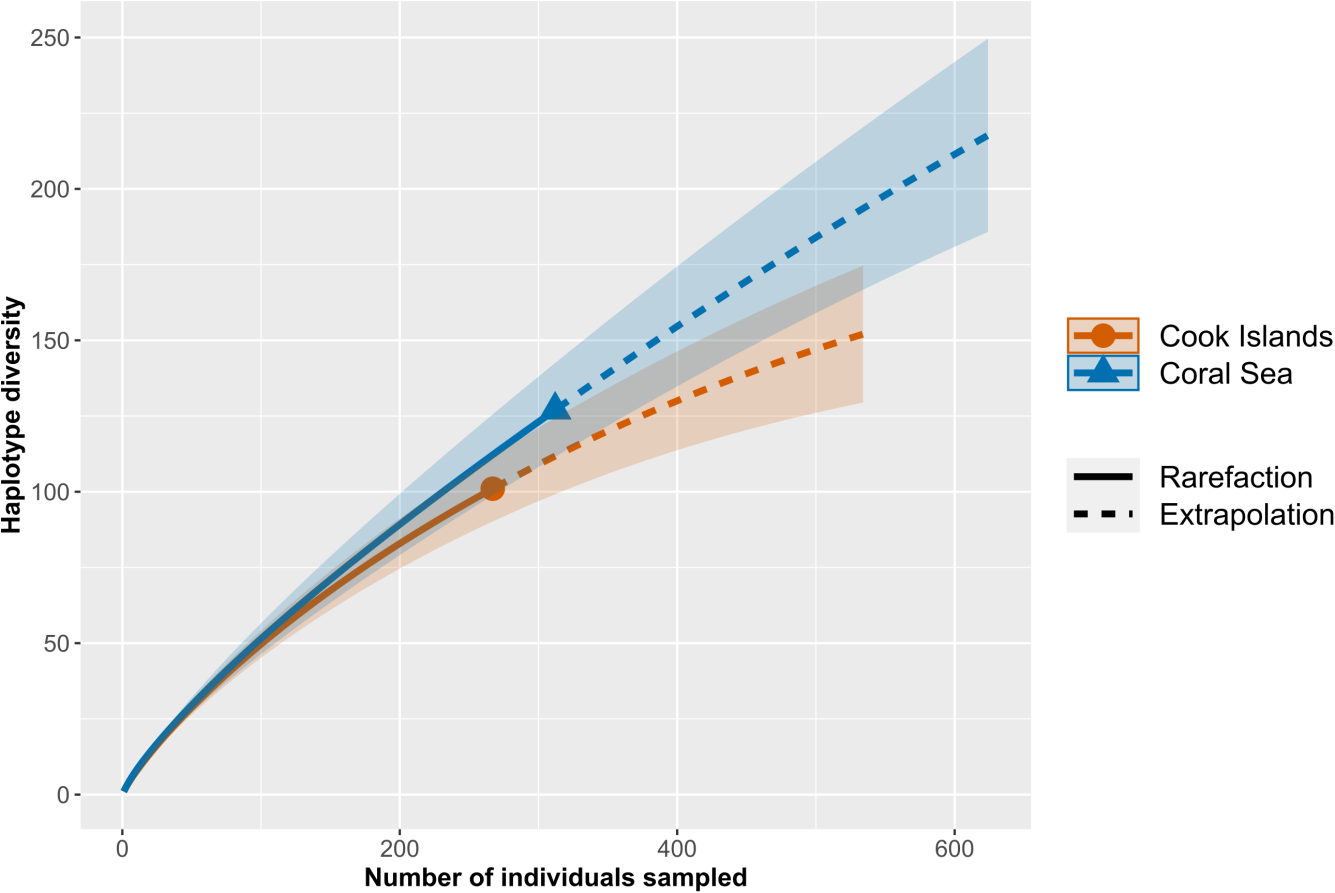
Haplotype rarefaction and extrapolation curves for *Tridacna maxima* sampled in the Coral Sea and Cook Islands. Shaded sections indicate the 95% CIs for each curve.

Haplotype R‐E curves revealed that much of the haplotype diversity within the Coral Sea and Cook Islands remains unsampled. The rarefaction curves for both regions displayed steep slopes and did not approach an asymptote (Figure 4). Extrapolation to a sample size of double the present sampling effort showed that this would have not been sufficient for characterising the complete genetic diversity in the two regions, although the curve for the Cook Islands appeared to be approaching an asymptote (Figure 4). The Chao1 index estimated the asymptote at 589.196 (±151.852 SE; 95% CI: 291.571–886.820) and 218.690 (±35.507 SE; 95% CI: 149.096–288.282) haplotypes for the Coral Sea and Cook Islands populations, respectively.

### 3.3 Population Expansion

**FIGURE 5:**
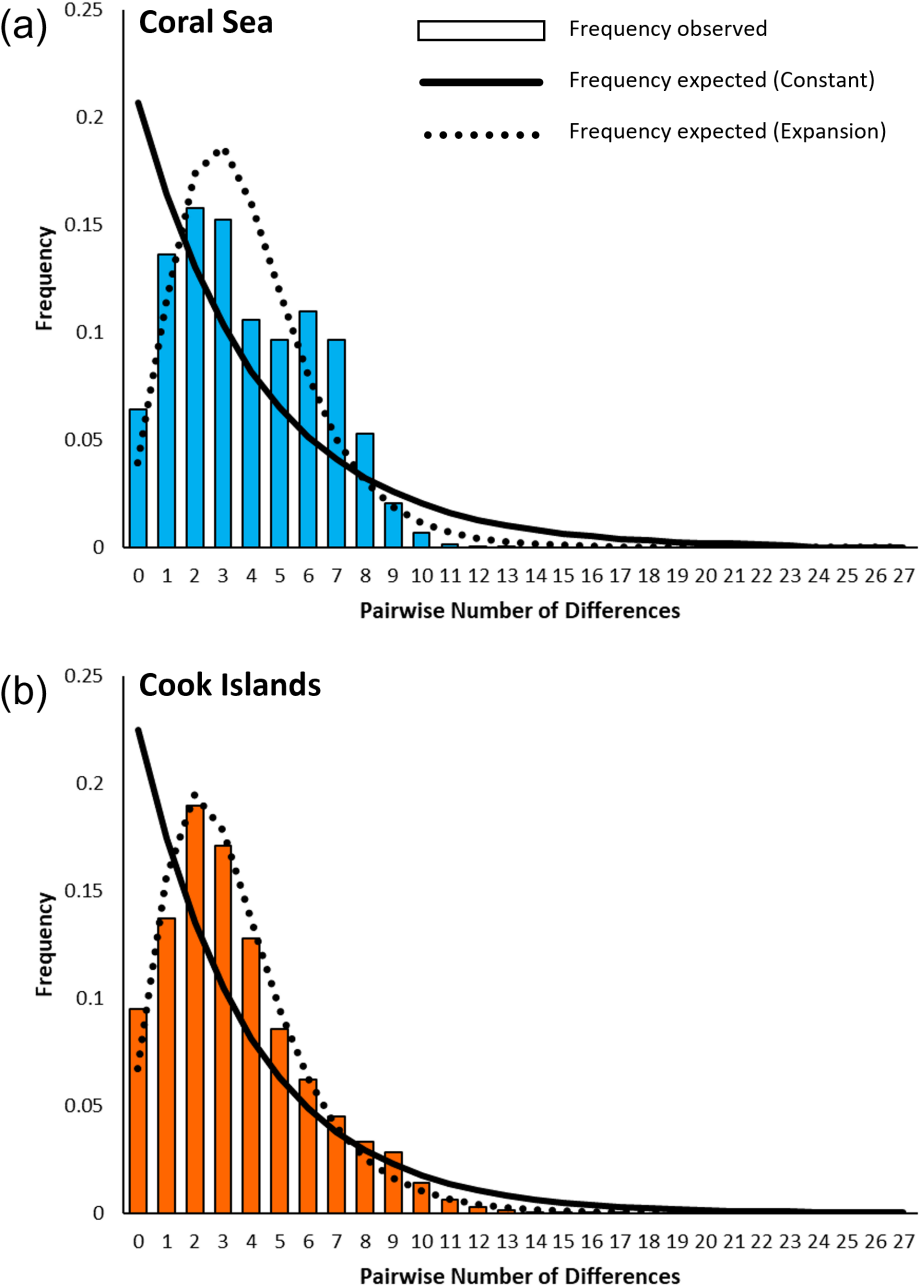
Mismatch distribution plots for *Tridacna maxima* sampled in the (a) Coral Sea and (b) Cook Islands. Frequency of the pairwise number of nucleotide differences between sampled individuals is represented by the coloured bars. Expected distributions under the constant population size model (solid black line) and population growth‐decline expansion model (dotted line) are fitted to the data.

Calculation of neutrality indices indicated population expansion for *T. maxima* from the Coral Sea and Cook Islands. For both regions, Tajima's *D* and Fu's *F*_S_ displayed significant negative values, whilst Ramos‐Onsins and Rozas' *R*_2_ was low and significant (Table 4). The mismatch distributions for both regions also showed a largely unimodal distribution which matched closely to the distribution expected for an expanding population under the population growth‐decline model (Figure 5). Nonetheless, Harpending's raggedness index, was also significant for both regions, suggesting that these data were not a good fit to the population growth‐decline model (Table 4). However, this metric has been shown to be the least powerful of the neutrality indices used here, especially with large sample sizes (Ramos‐Onsins and Rozas 2002). Given that most analyses (neutrality indices and mismatch distributions) provided evidence of population expansion, we conservatively suggest that expansion has occurred in *T. maxima* from both regions. Alternatively, given the obtained raggedness index, *T. maxima* populations in the Coral Sea and Cook Islands may have previously expanded and are now approaching a stable population size.

**TABLE 4:** Neutrality indices calculated for each region to assess population expansion in *Tridacna maxima*.

Region	*D*	*F*_S_	*R*_2_	H_RI_
Coral Sea	−2.1771**	−209.7180**	0.0230*	0.0114*
Cook Islands	−2.1525**	−32.8450**	0.0246*	0.0098*

Abbreviations: *D* = Tajima's *D*; *F*_S_ = Fu's *F*_S_ (tested at *α* = 0.02); H_RI_ = Harpending's raggedness index; *R*_2_ = Ramos‐Onsins and Rozas' *R*_2_. *p < 0.01; **p < 0.001.

### 3.4 Global Population Structure

**FIGURE 6:**
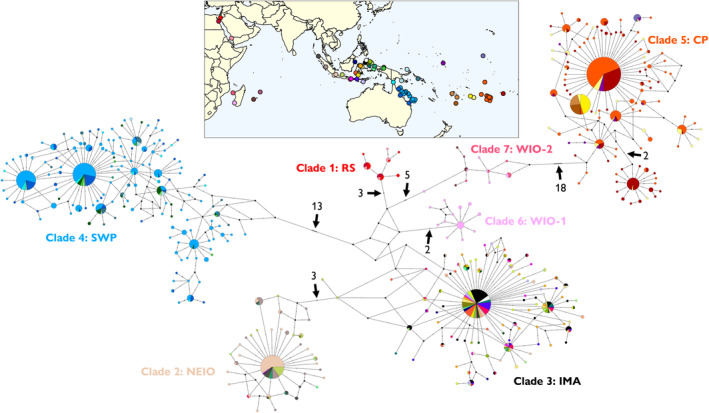
Median‐joining haplotype network for the global *Tridacna maxima* dataset, highlighting different haplogroups corresponding to mitochondrial clades across the distribution of the species. Circle size is proportional to the frequency of each haplotype and colour shading indicates the location where the haplotype was identified. Mutational steps between haplotypes are denoted by hatch marks and numbers. Small black dots represent hypothetical unsampled haplotypes. RS = Red Sea; NEIO = North‐Eastern Indian Ocean; IMA = Indo‐Malay Archipelago; SWP = South‐Western Pacific Ocean; CP = Central Pacific; WIO‐1 = Western Indian Ocean 1; WIO‐2 = Western Indian Ocean 2. Clade designations based on Keyse et al. (2018), Fauvelot et al. (2020) and Riquet et al. (2023).

A total of 1680 *T. maxima* sequences were assembled for the global dataset and encompassed sequences from all previously known clades. These sequences were collapsed into a total of 417 haplotypes, with the resulting haplotype network revealing the presence of seven distinct haplogroups corresponding to the seven previously identified mitochondrial clades (Figure 6). Individuals from the Coral Sea (light blue in Figure 6) were situated within the South‐Western Pacific clade, which also included individuals from the Great Barrier Reef, Torres Strait, Solomon Islands, Papua New Guinea and some sites in eastern Indonesia. This haplogroup was separated from the nearest haplogroup by a minimum of 13 mutations.

Individuals from the Cook Islands (orange in Figure 6) formed part of the distinct Central Pacific clade, which also comprised individuals from French Polynesia, Niue, Beveridge Reef, Fiji, Minerva Reefs (Tonga), Palmyra Atoll and Tarawa Atoll (Kiribati). This haplogroup was separated in the network from the nearest haplogroup by a minimum of 18 mutations. A sub‐group separated from the main Central Pacific haplogroup by at least two mutations and several unsampled haplotypes was also identified, which contained individuals predominantly from French Polynesia and a few individuals from the Cook Islands and Beveridge Reef. Three individuals from sites within the Central Pacific, namely Tarawa Atoll (*n* = 2) and Fiji (*n* = 1), possessed haplotypes that belonged to the Indo‐Malay Archipelago clade. All three individuals had the same haplotype, which was the most frequently occurring haplotype in the Indo‐Malay Archipelago clade (the large multi‐coloured circle in Figure 6).

Net average genetic distance between the seven mitochondrial clades ranged from 2.00% ± 0.75% to 8.76% ± 1.47% (Table S2). The Central Pacific clade displayed similar levels of divergence from all other clades, ranging from 7.34 (Clade 7) to 8.76% (Clade 6). When the sub‐group in the Central Pacific was treated as a separate grouping, net average distance ranged from 2.00% ± 0.74% to 8.98% ± 1.61%. The net average distance between the main Central Pacific clade (Clade 5a) and the sub‐group (Clade 5b) was 2.02% ± 0.85% (Table S2).

**FIGURE 7:**
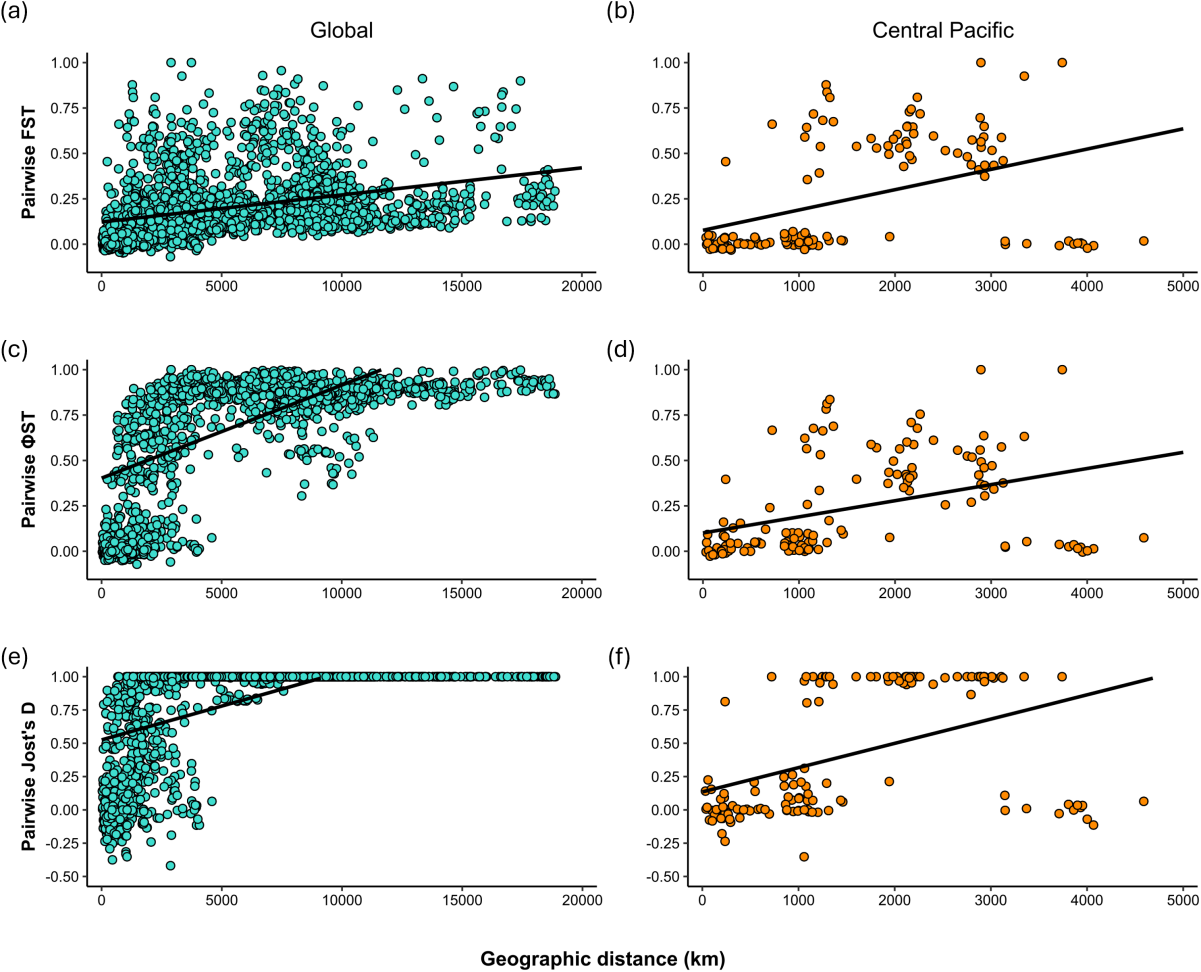
Isolation‐by‐distance plots for *Tridacna maxima* using three genetic differentiation metrics (*F*_ST_, *Φ*_ST_ and Jost's D) for the global dataset (a, c and e) and only sites within the Central Pacific (b, d and f).

Significant isolation‐by‐distance (IBD) was detected in *T. maxima* at both the global scale and within the Central Pacific. For the global dataset, Mantel tests revealed a significant relationship between the geographic and genetic distance matrices for all three tested metrics, whilst the permutation tests showed a significant positive correlation between geographic and genetic distance (Table 5; Figure 7a,c,e). The *Φ*_ST_ metric had the strongest correlation (Table 5). Many sites within 5000 km of each other showed very high levels of genetic differentiation, particularly for *Φ*_ST_ and Jost's D (Figure 7c,e). For the Central Pacific subset, Mantel tests and permutation tests for correlation were also significant, with Jost's D having the strongest correlation of the three metrics (Table 5; Figure 7b,d,f). Some sites separated by at least 1000 km displayed high levels of genetic differentiation, whereas others showed little to no genetic differentiation at distances up to ~4500 km (Figure 7b,d,f). This was consistent across the three metrics. The observed differences in levels of genetic differentiation at relatively small and large geographic distances across the Central Pacific are likely to have been driven by pairwise comparisons involving one or a few of the tested sites.

**TABLE 5:** Summary of Mantel and permutation tests for isolation‐by‐distance (IBD) in *Tridacna maxima* with three different genetics metrics.

Dataset	Metric	Mantel *r*	*p*	Correlation coefficient (*R* ^2^)	*p*
Global	*F*_ST_	0.337	0.001	0.114	< 0.0001
	*Φ*_ST_	0.602	0.001	0.363	< 0.0001
	Jost's D	0.558	0.001	0.312	< 0.0001
Central Pacific	*F*_ST_	0.426	0.034	0.182	< 0.0001
	*Φ*_ST_	0.391	0.043	0.153	< 0.0001
	Jost's D	0.448	0.026	0.201	< 0.0001

## 4 Discussion

This study assessed the genetic diversity and population structure of *Tridacna maxima* in Australia's Coral Sea Marine Park and the Cook Islands. Using mitochondrial DNA data, we show that *T. maxima* populations in the Coral Sea have no significant genetic differentiation across the ~1300 km sampling area. Conversely, population structure for *T. maxima* within the Cook Islands archipelago was detected, with significant differentiation amongst some islands ranging in distance from ~100 to 1300 km. Both regions had high haplotype diversities and showed evidence of population expansion. *Tridacna maxima* from the Coral Sea possessed mitochondrial haplotypes corresponding to the clade in the South‐Western Pacific, whilst those from the Cook Islands possessed haplotypes belonging to a clade unique to the Central Pacific.

Although the distance between the northernmost and southernmost sites sampled in the two regions were similar (~1300 km), different genetic structures were detected for *T. maxima* in the Coral Sea and Cook Islands. In the Coral Sea, our results indicated panmixia for *T. maxima* across the region, likely due to the maintenance of some gene flow amongst reefs over long periods. These results are consistent with previous allozyme work in the region, which showed no evidence of population structure for *T. maxima* amongst reefs on the Queensland Plateau and sites on the Great Barrier Reef with *F*_ST_ (Benzie and Williams 1992). Seven out of the eight reefs on the Queensland Plateau sampled by Benzie and Williams (1992) were also sampled in this study (i.e. Osprey, Bougainville, Holmes, Willis, Flinders, Chilcott and Lihou). A lack of population structure for the species has also been identified across the 2000 km stretch of the Red Sea (Lim et al. 2020). For the Cook Islands, however, weak but significant structure was detected in the archipelago. Here, most pairwise comparisons involving Manihiki, the only island from the northern group sampled in this study, were significant, as were comparisons for Palmerston (a centrally located island between the northern and southern groups) and the southern islands, Rarotonga and Mangaia.

The observed differences in genetic structure for *T. maxima* in the two regions likely relate to their geography. Previous allozyme work suggests that *T. maxima* is capable of extensive gene flow over large distances with a neighbourhood size of approximately 5000 km; however, this is only the case within island chains (Benzie and Williams 1997). The reefs in the Coral Sea comprise a relatively continuous chain with a maximum of 200 km between adjacent reefs, despite being separated by deep water (up to ~2 km) (Davies et al. 1989; Benzie and Williams 1992). The relatively close proximity of adjacent coral reefs may facilitate connectivity throughout the region. Connectivity is also likely aided by the movement of currents along Australia's east coast. Based on mitochondrial DNA markers, it has been shown that even low levels of connectivity (gene flow) over time preclude the genetic differentiation of populations, and this is particularly the case for species with high genetic diversity and large effective population sizes (Waples 1998; Hellberg et al. 2002).

In contrast, the Cook Islands comprises two groups of island chains that are separated by a wide section of the Pacific Ocean. This stretch of open ocean could limit dispersal of clam larvae between the northern and southern groups of the archipelago. Additionally, water depths separating the islands both within and between the island groups can reach ~5 km (Summerhayes 1967; Browne, Parianos, and Murphy 2023). Modelling of coral larval dispersal shows that the southern Cook Islands are isolated from other parts of the Pacific, including the northern Cook Islands, even when a larval duration of 60 days is considered (Treml et al. 2008). The pelagic larval period for *T. maxima* is 11 days (max. 19 days) (Jameson 1976) and measurement of larval swimming speed in *T. squamosa* suggests that giant clam larvae have limited horizontal swimming ability (Neo et al. 2015b). These factors therefore hamper dispersal potential. For islands within the southern group, the significant genetic structure detected even between relatively close islands could be due to a combination of small sample size for some sites (*n* < 10 for Mangaia and Mitiaro) and local currents and/or water depth restricting larval dispersal. Significant genetic structure for *T. maxima* between islands over small geographic scales has been observed in the Comoros Islands, Western Indian Ocean (Ahmed Mohamed et al. 2016). To explore population structure within the Cook Islands further, it is recommended that nuclear markers such as single nucleotide polymorphisms (SNPs) are applied to resolve any fine‐scale structure and assess gene flow between islands. Such work is currently underway (Liggins and Carvajal 2021). These analyses could also be supplemented with additional samples from previously unsampled islands in the northern group and increased replication from islands with smaller sample sizes.

Calculation of genetic diversity metrics showed that *T. maxima* populations in the Coral Sea and Cook Islands display high overall genetic diversity. Haplotype diversity for the two regions was greater than 0.90 (Table 1), indicating that these regions are important repositories of genetic diversity for the species, particularly in relation to their respective clades. Rarefaction analysis also suggests that these regions harbour a large number of haplotypes that were not sampled in our study. This was particularly the case for the Coral Sea population, whose extrapolation curve did not approach an asymptote when a doubled sampling effort was considered (Figure 4). This shows that extensive sampling is required to fully characterise the extant genetic diversity of *T. maxima* within these regions.

Whilst the finding of a similar level of genetic diversity between a legally harvested (Cook Islands) and legally protected (Coral Sea) population of *T. maxima* is encouraging, this high haplotype diversity may be a general feature of the species. Previous studies using the CO1 marker in other parts of *T. maxima*'s distribution have also documented large numbers of unique haplotypes as a proportion of sampling effort (Nuryanto and Kochzius 2009; DeBoer et al. 2014; Hui et al. 2016; Neo et al. 2018; Lim et al. 2020). This suggests that *T. maxima* displays mitochondrial hyperdiversity (Fourdrilis et al. 2016). High haplotype diversity has also been reported for other giant clam species, including *T. crocea* and *T. squamosa* (DeBoer et al. 2014; Hui et al. 2016), with mitochondrial hyperdiversity possibly present in *T. crocea* as well (Fourdrilis et al. 2016).

The high genetic diversity observed in *T. maxima* and other tridacnids is consistent with the pattern of generally high genetic diversity in marine species. High haplotype diversity has been documented in many species of mollusc, such as periwinkles (family Littorinidae), as well as other invertebrate species (e.g. echinoderms, cnidarians, arthropods and annelids) (Fourdrilis et al. 2016) and vertebrates (fishes and marine mammals) (Grant and Bowen 1998; Thompson et al. 2016; Robalo et al. 2020; Francisco et al. 2021). Whether this high diversity also reflects the regions sampled in this study is difficult to address as there are few mitochondrial DNA studies sampling from multiple reefs within the Coral Sea and limited population genetics studies within the Cook Islands. Most studies that involve the Cook Islands only sample from one or two islands and examine connectivity from a broad phylogeographic perspective. However, for species inhabiting the Coral Sea, the level of haplotype diversity seems to vary. Previous studies on sea cucumbers and damselfish have shown equally high haplotype diversities (Planes, Doherty, and Bernardi 2001; Uthicke and Benzie 2003), whereas work on nautilus and a scleractinian coral has revealed moderate to very low haplotype diversities, respectively (Sinclair et al. 2007; Klueter and Andreakis 2013). Although individual metrics for the sampled islands are not reported, two species of *Gnatholepis* goby in the Cook Islands also display a high overall haplotype diversity (Thacker 2004).

Our results confirm the presence of a distinct mitochondrial clade occurring in the Central Pacific Ocean and refine its distribution. Additional genetic data from the Central Pacific showed that this clade extends from Fiji in the west to French Polynesia in the east and the atolls of Palmyra and Tarawa in the north. These results support previous allozyme work for a distinct Central Pacific group (Benzie and Williams 1997) and the proposed extent of coverage by Riquet et al. (2023). The Central Pacific could be considered to encompass two clades, with the net average genetic distance between Clade 5a and Clade 5b (2.02%) being very close to the distance between the Red Sea (Clade 1) and Western Indian Ocean (Clade 6) clades (2.00%). Further refinement of clade boundaries and identifying possible gene flow from other clades, such as the Indo‐Malay Archipelago or South‐Western Pacific clades, could be achieved through sequencing of CO1 from locations such as Vanuatu, New Caledonia, the Marshall Islands, Guam and Palau. Such sampling may be aided by extracting DNA from preserved clam tissue or shell available at markets (Gardner et al. 2012) instead of collecting tissue from live individuals.

The results from this study have important implications for management and conservation of *T. maxima* populations. Whilst genetic diversity in both regions was largely spread across the sampling areas, the difference in population structure suggests that separate management strategies should be used for clams in the Coral Sea and Cook Islands. The lack of population structure detected for *T. maxima* in the sampled area of the Coral Sea suggests that the species could be considered as one population for the purposes of management and monitoring within the marine park. Additional genetic data using nuclear markers (microsatellites or SNPs) would be required to confirm the existence of a single *T. maxima* population within the marine park and infer more recent contemporary gene flow amongst reefs. Nuclear data could also be used to infer gene flow with nearby countries outside the marine park, such as the Solomon Islands, Papua New Guinea, New Caledonia and Vanuatu. For the Cook Islands, separate management and monitoring of *T. maxima* in the northern and southern groups is suggested given the significant population structure identified. Moreover, the genetic diversity metrics documented here could serve as a baseline measurement for future reassessment of genetic diversity in these populations, such as after natural disasters (e.g. cyclones), the introduction of new harvesting regimes or potential restoration projects, and for routine monitoring over time. Any major deviations in genetic diversity from this baseline could indicate the effectiveness of management actions.

Conservation and management information can also be derived from the global assessment of *T. maxima* clades. The confirmation and refined distribution of the Central Pacific clade means that the origin of illegally traded clam products can be more accurately attributed to a specific region, possibly a country, using genetic tools. Furthermore, the distinctiveness of the Central Pacific clade indicates that any restocking of *T. maxima* populations within this region should only use individuals from this clade to avoid potential outbreeding depression. Finally, our results show that the proposed cryptic species or operational taxonomic unit (OTU) identified by Liu et al. (2020) in Cenderawasih Bay (Indonesia) is not confined to this location. This is because haplotypes from Cenderawasih Bay were shared by clams from the Coral Sea and several other locations in the South‐Western Pacific (Huelsken et al. 2013; Keyse et al. 2018).

**FIGURE A2:**
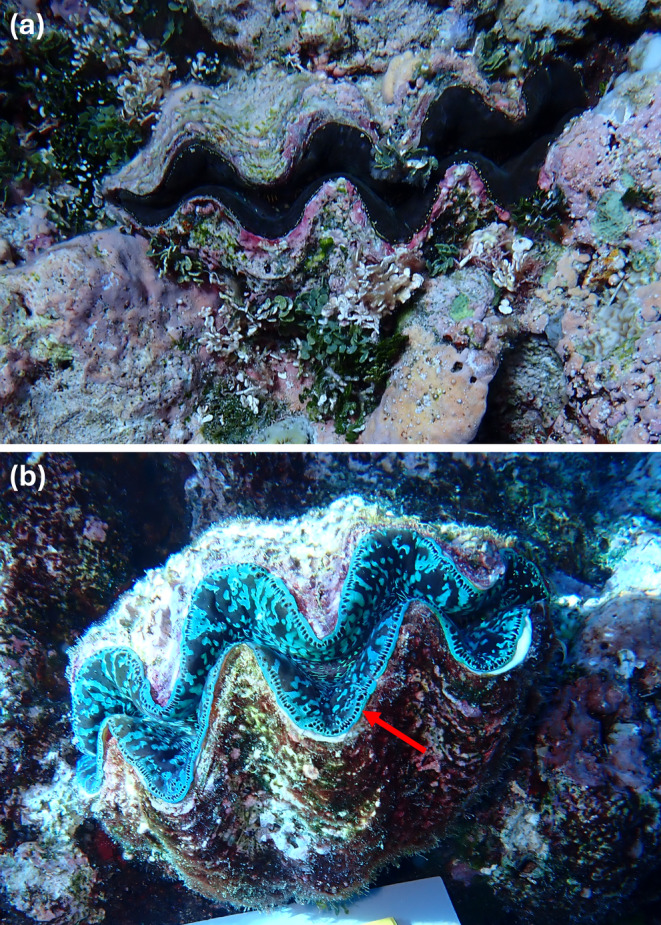
(a) Example of a giant clam (diameter 190 mm) from Herald Cays (Coral Sea) assigned as either *Tridacna maxima* or *Tridacna squamosa* based on photographs. Note the amount of encrusting material and presence of epibionts on the shell and the dark mantle colouration, making diagnostic features difficult to identify. Individual was genetically confirmed as *T. maxima*; (b) Genetically confirmed *T. maxima* (diameter 200 mm) on Bougainville Reef (Coral Sea) displaying the characteristic regular arrangement of hyaline organs (red arrow) around the edge of the mantle. Photo credits: Peter C. Doll.

**FIGURE A3:**
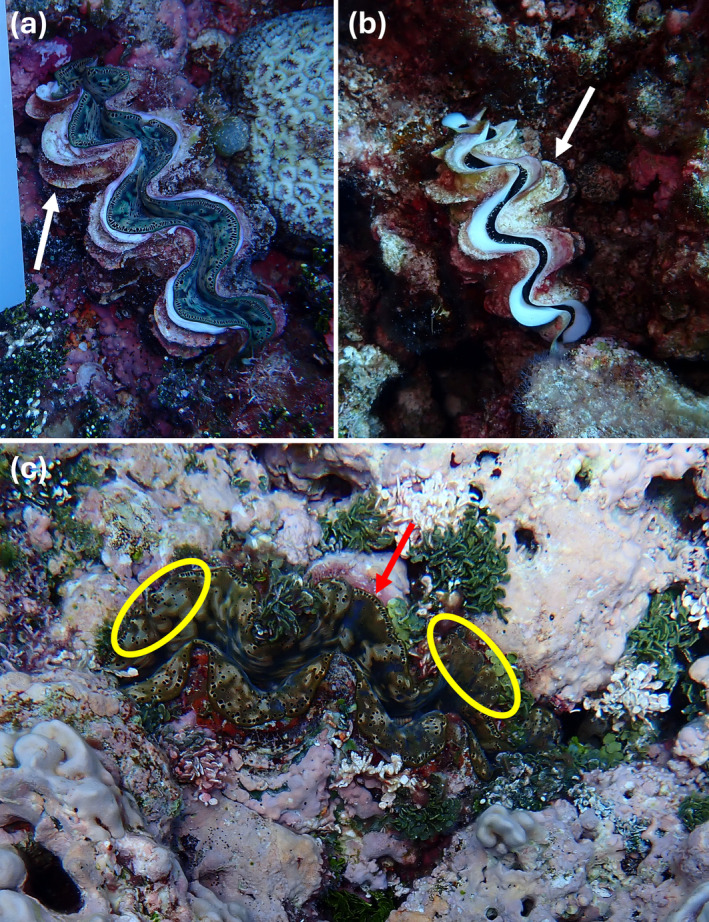
Two genetically confirmed Coral Sea *Tridacna maxima* from (a) Herald Cays (diameter 180 mm) and (b) Lihou Reef (diameter 200 mm) displaying prominent scutes (white arrows) on their shells. (c) Genetically confirmed *T*. *maxima* (diameter 160 mm) on Willis Reef (Coral Sea) displaying an irregular (yellow circles) and regular (red arrow) arrangement of hyaline organs on its mantle. Photo credits: Peter C. Doll.

The mismatch between field identification and molecular identity observed here highlights a potential issue with distinguishing between *T. maxima* and *T. squamosa* in the Coral Sea. Key morphological features that delineate species [see Rosewater 1965; Lucas 1988; Norton and Jones 1992], such as the size of the byssal orifice and the size and positioning of scutes, were generally unable to be reliably used for these photographs. This is because the byssal orifice is underneath the clam and, thus, cannot be seen, and the shells were often covered in encrusting material or epibionts which obscured the scutes (Figures A1, A2, A3). Differentiating between *T. maxima* and *T. squamosa* based on scutes alone may also not be entirely useful as *T. maxima* can display large, prominent scutes that are similar to those of *T. squamosa* (Su et al. 2014) (Figure A3a,b), whilst *T. squamosa* can possess closely spaced scutes characteristic of *T. maxima* (Pappas et al. 2017). Other morphological features such as the presence and shape of tentacles in the incurrent siphon were also not always easily visible. Although the habitat ecology of *T. maxima* and *T. squamosa* can allow for differentiation of species, with the former typically partially embedded in the reef framework and the latter free‐living (Neo et al. 2017, 2019), applying this as a strict rule may lead to misidentification. *Tridacna squamosa* has been observed nestled or embedded into the reef (Figure A1) (Pappas et al. 2017), whilst *T. maxima* can be free‐living on the reef surface (Figure A2b) (Pappas et al. 2017).

Here, the most reliable morphological feature that corresponded to the genetic species identity was the arrangement of the hyaline organs (Figure A2b), which in *T. maxima* is generally a continuous line around the edge of the mantle (Rosewater 1965; Neo et al. 2017). The hyaline organs could, however, sometimes be difficult to identify in clams with dark‐coloured mantles (Figure A2a), and this feature can be variable with some *T. maxima* displaying an irregular arrangement (Figure A3c) (Ramesh et al. 2017). Nevertheless, we recommend that the arrangement of the hyaline organs be used to identify *T. maxima* in any future surveys or citizen science programs conducted in the Coral Sea.

In conclusion, mitochondrial DNA analysis of *T. maxima* from the Coral Sea and Cook Islands has demonstrated that these two regions contain clam populations with high haplotype diversity and are important repositories of genetic diversity for this species. The molecular data generated here will assist with the conservation and management of *T. maxima* in these regions to ensure that populations are adequately protected, and legal harvesting is sustainable.

## Author Contributions

**Ryan J. Nevatte:** conceptualization (equal), data curation (equal), formal analysis (equal), funding acquisition (equal), investigation (equal), methodology (equal), visualization (equal), writing – original draft (lead), writing – review and editing (equal). **Michael R. Gillings:** conceptualization (equal), data curation (equal), formal analysis (equal), funding acquisition (equal), investigation (equal), methodology (equal), resources (equal), writing – review and editing (equal). **Kirby Morejohn:** conceptualization (equal), funding acquisition (equal), resources (equal), writing – review and editing (equal). **Lara Ainley:** conceptualization (equal), funding acquisition (equal), resources (equal), writing – review and editing (equal). **Libby Liggins:** conceptualization (equal), data curation (equal), methodology (equal), resources (equal), writing – review and editing (equal). **Morgan S. Pratchett:** conceptualization (equal), resources (equal), writing – review and editing (equal). **Andrew S. Hoey:** conceptualization (equal), resources (equal), writing – review and editing (equal). **Peter C. Doll:** conceptualization (equal), resources (equal), writing – review and editing (equal). **Brendon Pasisi:** conceptualization (equal), funding acquisition (equal), resources (equal), writing – review and editing (equal). **Jane E. Williamson:** conceptualization (equal), data curation (equal), formal analysis (equal), funding acquisition (equal), investigation (equal), methodology (equal), writing – review and editing (equal).

## Conflicts of Interest

The authors declare no conflicts of interest.

## Supporting information


**File S1.** Excel file with a list of all sequences used in the global analysis.

**File S2.** DnaSP formatted NEXUS file of sequences used in the global analysis.

**Figure S1.** Median‐joining haplotype network for *T. maxima* samples collected from the Coral Sea and Cook Islands.
**Figure S2.** Median‐joining haplotype network for *T. maxima* from the Coral Sea based on only the first and second codon positions.
**Figure S3.** Median‐joining haplotype network for *T. maxima* from the Cook Islands based on only the first and second codon positions.
**Table S1.** Pairwise *F*_ST_ and *Φ*_ST_ matrix for *Tridacna maxima* from reefs in the Coral Sea.
**Table S2.** Net average genetic distance for *T. maxima* clades.
**Text S1** Explanation of supplementary files.

## Data Availability

All new genetic data generated/used for this study have been deposited in GenBank [Accession numbers: PQ146647—PQ146887 (Coral Sea and Cook Islands) and PQ339677—PQ339813 (Niue, Beveridge Reef, Minerva Reef and Fiji)], and metadata uploaded to the Genomic Observatories Metadatabase (GEOME, Deck et al. (2017); Riginos et al. (2020) and accessioned as Tmaxima_CO1_Nevatte at http://n2t.net/ark:/21547/FpI2). The distribution of haplotypes amongst the sampled reefs/islands of the Coral Sea and Cook Islands is presented in the Supporting Information (Tables S3–S5). A list of all sequences used in the global analysis (File S1) and a DnaSP formatted NEXUS file (File S2) are also provided (see Text S1 in the Supporting Information document for additional details).
