# Supplementary material for: Of Clams and Clades: Genetic Diversity and Connectivity of Small Giant Clams (Tridacna maxima) in the Southern Pacific Ocean
Source: Ecol Evol. 2024 Oct 25;14(10):e70474. doi: 10.1002/ece3.70474 (PMC11511625; doi:10.1002/ece3.70474)
Supplement: Supplementary file 3 — Figure S1. Median‐joining haplotype network for T. maxima samples collected from the Coral Sea and Cook Islands. Figure S2. Median‐joining haplotype network for T. maxima from the Coral Sea based on only the first and second codon positions. Figure S3. Median‐joining haplotype network for T. maxima from the Cook Islands based on only the first and second codon positions. Table S1. Pairwise F ST and Φ ST matrix for Tridacna maxima from reefs in the Coral Sea. Table S2. Net average genetic distance for T. maxima clades. Text S1 Explanation of supplementary files. [file ECE3-14-e70474-s001.zip › Nevatte et al. - South Pacific Tridacna maxima - Supplementary material V2.docx]

**Supplementary Material:**

Of clams and clades: genetic diversity and connectivity of small giant clams (*Tridacna maxima*) in the southern Pacific Ocean

*Ecology and Evolution*

**Authors:**

Ryan J. Nevatte* | Michael R. Gillings | Kirby Morejohn | Lara Ainley | Libby Liggins | Morgan S. Pratchett | Andrew S. Hoey | Peter C. Doll | Brendon Pasisi | Jane E. Williamson

* Corresponding author: rjnevatte@gmail.com

School of Natural Sciences, Macquarie University, New South Wales, 2109, Australia

**Table of Contents:**

| **Table S1:** Pairwise *F*_ST_ and Φ_ST_ matrix for *Tridacna maxima* from reefs in the Coral Sea. | Page 2 |
| --- | --- |
| **Table S2:** Net average genetic distance for *T. maxima* clades. | Page 3 |
| **Figure S1:** Median-joining haplotype network for *T. maxima* samples collected from the Coral Sea and Cook Islands. | Page 4 |
| **Figure S2:** Median-joining haplotype network for *T. maxima* from the Coral Sea based on only the first and second codon positions. | Page 5 |
| **Figure S3:** Median-joining haplotype network for *T. maxima* from the Cook Islands based on only the first and second codon positions. | Page 6 |
| **Supplementary text:** Explanation of supplementary files | Page 7 |

Table S1: Pairwise *F*_ST_ values (lower diagonal) and Φ_ST_ values (upper diagonal) for *Tridacna maxima* samples collected from reefs in the Coral Sea. Values in italics were significant at α = 0.05 prior to correction for multiple comparisons. HRN = Heron Island; SAU = Saumarez Reef; WRE = Wreck Reef; KEN = Kenn Reef; FRE = Frederick Reef; MAR = Marion Reef; LIH = Lihou Reef; CHI = Chilcott Reef; WIL = Willis Reef; HER = Herald Cay; FLI = Flinders Reef; HOL = Holmes Reef; BOU = Bougainville Reef; OSP = Osprey Reef.

|  | **HRN** | **SAU** | **WRE** | **KEN** | **FRE** | **MAR** | **LIH** | **CHI** | **WIL** | **HER** | **FLI** | **HOL** | **BOU** | **OSP** |
| --- | --- | --- | --- | --- | --- | --- | --- | --- | --- | --- | --- | --- | --- | --- |
| **HRN** | - | -0.0610 | -0.0152 | -0.0257 | -0.0318 | 0.2142 | -0.0561 | 0.1149 | -0.0081 | -0.0626 | -0.0004 | -0.0796 | -0.0451 | -0.0196 |
| **SAU** | 0.0269 | - | 0.0276 | 0.0184 | 0.0174 | 0.1980 | -0.0213 | *0.1562* | 0.0171 | -0.0004 | *0.0562* | -0.0452 | 0.0008 | 0.0270 |
| **WRE** | 0.0295 | -0.0064 | - | -0.0161 | -0.0209 | -0.0688 | 0.0199 | 0.0164 | -0.0067 | 0.0011 | -0.0164 | -0.0273 | -0.0077 | -0.0148 |
| **KEN** | 0.0415 | -0.0149 | -0.0126 | - | -0.0189 | -0.0422 | 0.0121 | 0.0128 | -0.0048 | -0.0058 | -0.0156 | -0.0378 | -0.0152 | -0.0139 |
| **FRE** | 0.0630 | 0.0108 | -0.0124 | -0.0134 | - | 0.0126 | 0.0022 | 0.0492 | 0.0058 | -0.0128 | -0.0132 | -0.0556 | -0.0214 | -0.0140 |
| **MAR** | 0.2116 | 0.0210 | 0.0223 | 0.0156 | 0.0439 | - | 0.1995 | -0.2403 | 0.0079 | 0.0592 | -0.1347 | 0.1151 | 0.0148 | -0.0583 |
| **LIH** | 0.0388 | -0.0179 | -0.0092 | -0.0126 | -0.0107 | 0.0177 | - | *0.1509* | 0.0209 | -0.0087 | *0.0508* | -0.0396 | -0.0013 | 0.0221 |
| **CHI** | *0.2030* | *0.0661* | *0.0598* | *0.0477* | *0.0650* | -0.1340 | *0.0526* | - | 0.0561 | *0.0808* | -0.0172 | 0.0748 | 0.0539 | 0.0191 |
| **WIL** | *0.1002* | -0.0040 | -0.0001 | -0.0076 | 0.0019 | 0.0124 | -0.0012 | 0.0387 | - | 0.0096 | 0.0025 | -0.0038 | -0.0061 | -0.0076 |
| **HER** | *0.8701* | 0 | 0.0052 | -0.0020 | 0.0032 | 0.0352 | 0.0005 | *0.0658* | -0.0050 | - | 0.0154 | -0.0424 | -0.0139 | 0.0029 |
| **FLI** | 0.0386 | -0.0058 | -0.0133 | -0.0121 | -0.0108 | 0.0342 | -0.0109 | *0.0612* | -0.0041 | -0.0023 | - | -0.0182 | -0.0022 | -0.0125 |
| **HOL** | -0.0539 | -0.0153 | -0.0218 | -0.0142 | -0.0090 | 0.1028 | -0.0124 | *0.1253* | 0.0260 | 0.0196 | -0.0231 | - | -0.0516 | 0.0229 |
| **BOU** | 0.0282 | -0.0129 | -0.0106 | -0.0132 | -0.0049 | 0.0449 | -0.0092 | *0.0759* | 0.0013 | -0.0021 | -0.0149 | -0.0217 | - | -0.0117 |
| **OSP** | *0.0754* | -0.0106 | -0.0027 | -0.0034 | 0.0014 | 0.0010 | -0.0107 | *0.0446* | -0.0092 | -0.0096 | -0.0066 | 0.0136 | -0.0040 | - |

Table S2: Net average genetic distance (uncorrected P-distance) calculated for each clade identified in the global dataset of *Tridacna maxima*. Standard errors (from 100 bootstrap replicates) are shown in the upper diagonal.

| **Seven clades** | Clade  1 | Clade  2 | Clade  3 | Clade  4 | Clade  5 | Clade  6 | Clade  7 |  |
| --- | --- | --- | --- | --- | --- | --- | --- | --- |
| Clade 1 – Red Sea | - | 0.0101 | 0.0078 | 0.0144 | 0.0143 | 0.0075 | 0.0094 |  |
| Clade 2 – NE Indian Ocean | 0.0366 | - | 0.0084 | 0.0147 | 0.0145 | 0.0095 | 0.0100 |  |
| Clade 3 – IM Archipelago | 0.0274 | 0.0258 | - | 0.0134 | 0.0139 | 0.0076 | 0.0085 |  |
| Clade 4 – SW Pacific Ocean | 0.0724 | 0.0771 | 0.0694 | - | 0.0131 | 0.0136 | 0.0135 |  |
| Clade 5 – Central Pacific | 0.0833 | 0.0846 | 0.0776 | 0.0829 | - | 0.0147 | 0.0150 |  |
| Clade 6 – W Indian Ocean 1 | 0.0200 | 0.0303 | 0.0217 | 0.0734 | 0.0876 | - | 0.0099 |  |
| Clade 7 – W Indian Ocean 2 | 0.0310 | 0.0319 | 0.0245 | 0.0713 | 0.0734 | 0.0320 | - |  |
| **Two Central Pacific clades** | Clade  1 | Clade  2 | Clade  3 | Clade  4 | Clade 5a | Clade 5b | Clade  6 | Clade  7 |
| Clade 1 – Red Sea | - | 0.0104 | 0.0082 | 0.0142 | 0.0146 | 0.0147 | 0.0074 | 0.0095 |
| Clade 2 – NE Indian Ocean | 0.0366 | - | 0.0086 | 0.0147 | 0.0147 | 0.0161 | 0.0096 | 0.0102 |
| Clade 3 – IM Archipelago | 0.0274 | 0.0258 | - | 0.0132 | 0.0142 | 0.0142 | 0.0078 | 0.0086 |
| Clade 4 – SW Pacific Ocean | 0.0724 | 0.0771 | 0.0694 | - | 0.0132 | 0.0116 | 0.0135 | 0.0132 |
| Clade 5a – Central Pacific | 0.0852 | 0.0859 | 0.0790 | 0.0856 | - | 0.0085 | 0.0152 | 0.0155 |
| Clade 5b – Central Pacific | 0.0834 | 0.0898 | 0.0829 | 0.0741 | 0.0202 | - | 0.0149 | 0.0147 |
| Clade 6 – W Indian Ocean 1 | 0.0200 | 0.0303 | 0.0217 | 0.0734 | 0.0895 | 0.0870 | - | 0.0099 |
| Clade 7 – W Indian Ocean 2 | 0.0310 | 0.0319 | 0.0245 | 0.0713 | 0.0750 | 0.0766 | 0.0320 | - |


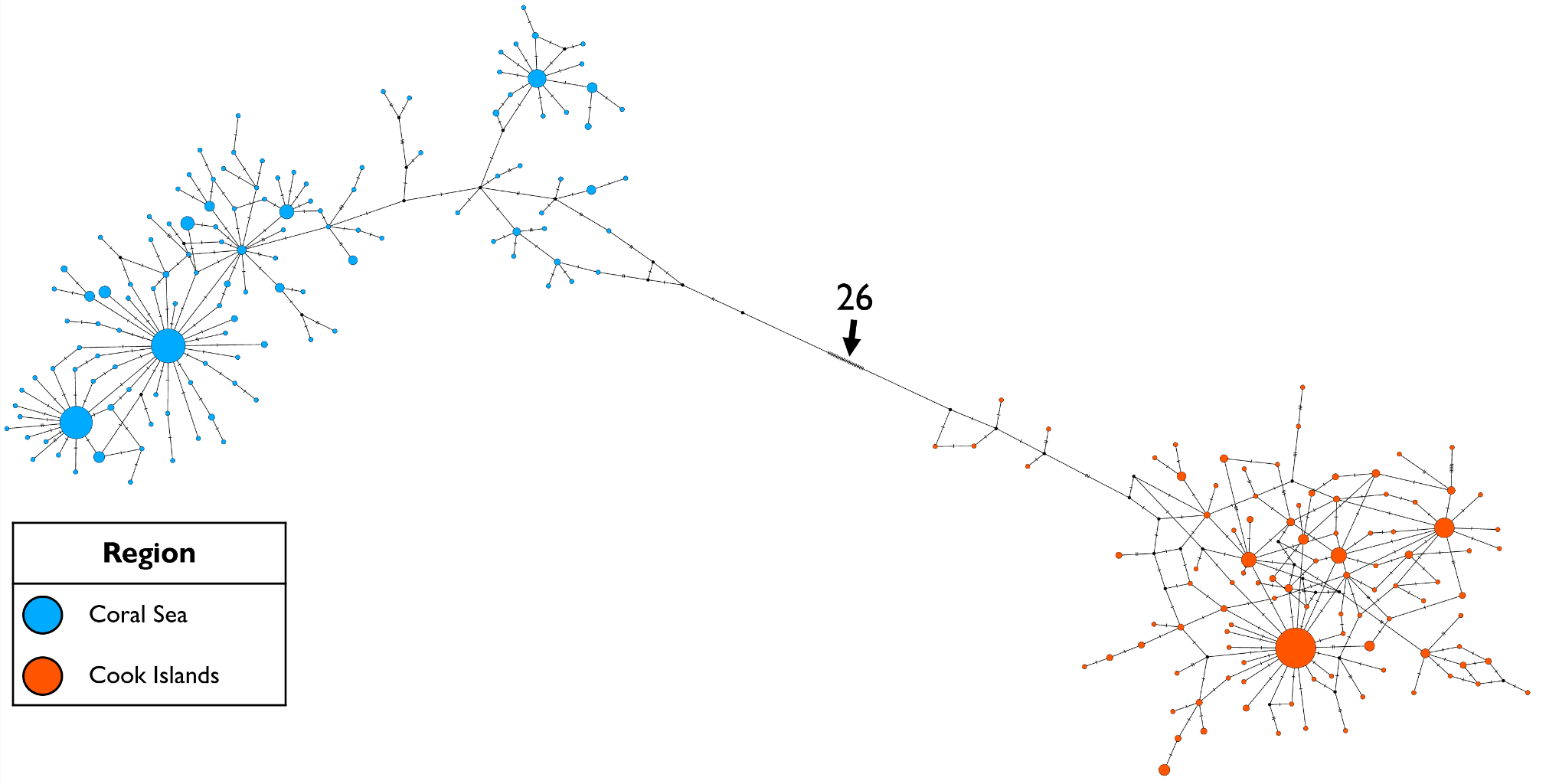


Figure S1: Median-joining haplotype network for *Tridacna maxima* collected from two regions of the South Pacific Ocean. Circle size is proportional to the frequency of each haplotype and colour shading indicates the region where the haplotype was identified. Mutational steps between haplotypes are denoted by hatch marks and small black dots represent hypothetical unsampled haplotypes.


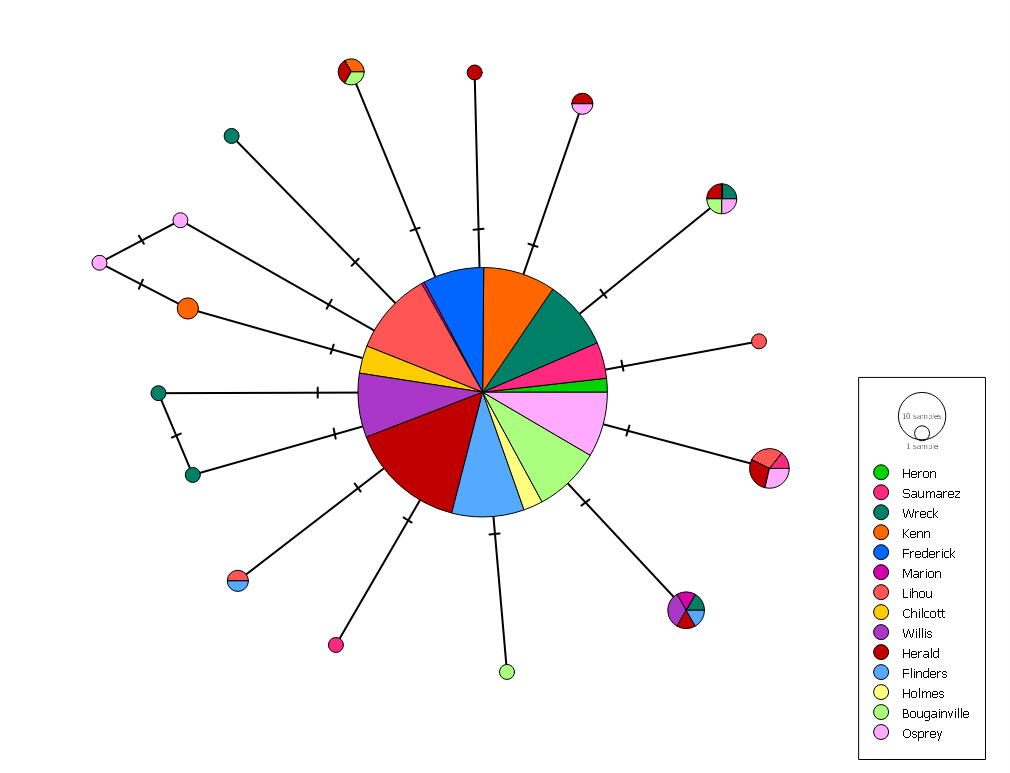


Figure S2: Median-joining haplotype network for *Tridacna maxima* from the Coral Sea based on only the first and second codon positions. Circle size is proportional to the frequency of each haplotype and colour shading indicates the site where the haplotype was identified. Mutational steps between haplotypes are denoted by hatch marks.


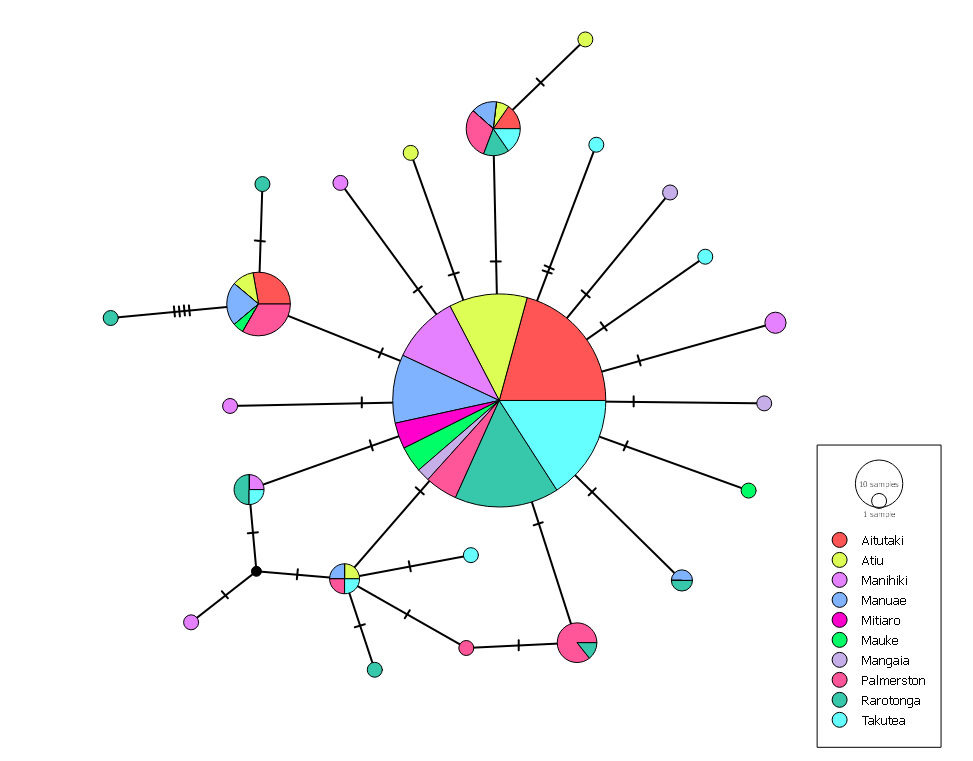


Figure S3: Median-joining haplotype network for *Tridacna maxima* from the Cook Islands based on only the first and second codon positions. Circle size is proportional to the frequency of each haplotype and colour shading indicates the site where the haplotype was identified. Mutational steps between haplotypes are denoted by hatch marks and small black dots represent hypothetical unsampled haplotypes.

**Supplementary text: Explanation of supplementary files**

The supplementary files for this manuscript include:

- An Excel file containing information on the sequences used in the global analysis of *Tridacna maxima*
- A DnaSP formatted NEXUS file containing all 1,680 sequences used in the global analysis (316 bp)

Additional details for these files are outlined below.

Excel file

*Tab 1: Tridacna maxima global seqs*

This provides details on each sequence used in the global analysis, including the grouping of sequences into populations (Population), where the sequence was obtained (Sequence Source) and the associated reference (Reference). Full details for each reference are provided in the Reference list of this paper. The Sequence ID corresponds to the TAXLABELS in the TAXA block of the DnaSP NEXUS file.

For sequences sourced from GEOME, a direct link to the page with the sequence is provided (Link to fasta from GEOME). Sequence IDs with cells highlighted in blue (e.g. MF167475.1**,** JX974933.1) indicate that the Sequence ID is also the GenBank Accession number. If the GenBank Accession (or Sequence ID for Palmyra and Kiribati) has an underscore and a number following it (e.g. MF167478.1_2, Kiribati_GD0024_8), this indicates that it is a duplicate of that sequence, with the total number corresponding to the frequency of occurrence from the literature.

For the purposes of constructing the haplotype network, sequences from each site in the Cook Islands (sequences starting with CI_) and the Coral Sea (sequences starting with CS_) were not treated as a separate populations and combined for each region (total of 267 sequences for Cook Islands and 307 sequences for Coral Sea). Sequences from Heron Island were grouped with other sequences from the Great Barrier Reef (GBR) (total of 76 sequences).

*Tab 2: GPS coords for sites*

This provides the latitudes and longitudes for each location/population used in the Isolation-by-Distance (IBD) analysis. Note: coordinates for Marion Reef, Bootless Bay – PNG and Mauritius are not provided since these sites only contained one or two individuals and were not used in the IBD analysis.

DnaSP formatted NEXUS file

This is a NEXUS file generated in DnaSP containing all 1,680 sequences used in the global analysis (316 bp). The names for each sequence match those stated in the Sequence ID column in the Excel file, however the DnaSP NEXUS only displays the first 20 characters. Each sequence has been assigned to the appropriate Population (TaxaSet) for the IBD analysis. This file can be opened in DnaSP and can be used to collapse sequences into haplotypes (see Methods section of this paper) and generate the files for analysis in Arlequin. Nucleotide positions with a question mark (?) represent an ambiguous base since DnaSP cannot read ambiguity codes.

Two files will be generated: 1) an Arlequin project file (*.arp) and 2) a haplotype file (*.hap). The .hap file contains the haplotypes used by the Arlequin project for analysis. Both the .arp and .hap files must be in the same directory for the analysis to work since the project will look for an external haplotype list. The file name of the haplotype list must match the file name listed in the project file. Check the [[HaplotypeDefinition]] HapList = EXTERN section of the project file to make sure the file names match if there are any problems with running the analysis in Arlequin.
